# Association of meteorological factors with allergic rhinitis: a systematic review and meta-analysis

**DOI:** 10.1186/s12889-025-26078-6

**Published:** 2025-12-30

**Authors:** Qingpeng Li, Wangyang Gu, Huike Feng, Yihao Xue, Linling Xu, Dan Wang, Shilu Tong, Guiyan Yang, Shijian Liu

**Affiliations:** 1 School of Public Health, Hainan Medical University, Haikou, China; 2 Sanya Women and Children’s Hospital Affiliated to Hainan Medical University, 339 Yingbin Road, Sanya, 572022 China; 3 Hainan Branch, Shanghai Children’s Medical Center, School of Medicine, Shanghai Jiao Tong University, Sanya, China; 4 School of Basic Medicine and Life Science, Hainan Medical University, Haikou, China; 5 School of Public Health and Social Work, Queensland University of Technology, Brisbane, Australia; 6 National Institute of Environmental Health, Chinese Centers for Disease Control and Prevention, Beijing, China; 7 Shanghai Children’s Medical Center, Shanghai Jiao Tong University, School of Medicine, Shanghai, China; 8 Clinical Research Unit, Shanghai Municipal Hospital of Traditional Chinese Medicine, Shanghai University of Traditional Chinese Medicine, Shanghai, China

**Keywords:** Meteorological factors, Allergic rhinitis, Temperature, Humidity, Precipitation

## Abstract

**Background:**

Associations of meteorological factors with allergic rhinitis (AR) have been examined in numerous prior research works, yet conclusions regarding these correlations remain inconsistent.

**Methods:**

Eligible articles were identified from PubMed, ProQuest, Scopus, Web of Science, and Embase databases by December 1, 2025. Five factors were analyzed: temperature, relative humidity, precipitation, atmospheric pressure, and wind speed. Random-effect models calculated pooled odds ratio (OR) and 95% confidence intervals (CI). Subgroup and sensitivity analysis explored heterogeneity, while publication bias was evaluated.

**Results:**

Twenty-two observational studies involving 5,218,796 participants were included. For temperature, a 1 ℃ increase corresponded to a 14% elevated AR risk [OR (95%CI) = 1.14 (1.03–1.25)], whereas subgroup analyses uncovered more significant associations in temperate zones, high-quality studies and medical record-based outcomes. For humidity, a 1% increase in relative humidity was correlated with a 4% reduced AR risk [OR (95%CI) = 0.96 (0.94–0.99)], with more pronounced protective effects in subtropical regions, middle-income countries and medical record-based outcomes. A 10 mm increase in precipitation was correlated with an 18% higher AR risk [OR (95%CI) = 1.18 (1.01–1.38)], though sensitivity analysis indicated the results were influenced by individual studies. There were no significant links between atmospheric pressure, wind speed and AR (*P* > 0.05). Additionally, no obvious publication bias was detected in this study.

**Conclusions:**

Temperature and precipitation may increase the risk of AR, while humidity has a protective effect, with the effects varying across climate zones, income levels, study quality and outcome type.

**Supplementary Information:**

The online version contains supplementary material available at 10.1186/s12889-025-26078-6.

## Introduction

Allergic rhinitis (AR) represents a prevalent inflammatory disease triggered via an immunoglobulin E (IgE)-mediated immune response to a diverse array of inhaled allergens [1]. Key clinical manifestations of AR include obstruction, sneezing, itching and runny nose, which are often accompanied by ocular pruritus, redness or watery eyes [2, 3]. These symptoms not only compromise quality of life but also contribute to comorbidities like sleep disruption and diminished work efficiency [4]. Recent research suggests that the prevalence of AR varies from 10% to 40% across various regions globally, with the highest prevalence in developed western countries [5, 6]. This high prevalence and its geographically uneven distribution impose a formidable socioeconomic burden, including both direct treatment costs and indirect expenses associated with healthcare [7, 8]. Despite its global public health impact, the multifactorial etiology of AR remains incompletely understood, as numerous interacting factors (e.g., genetics, epigenetics, lifestyle, and environmental exposures) contribute to this complexity [9–11].

Currently, amid escalating global climate shifts including global warming, altered precipitation regimes and a rising incidence of extreme meteorological events have become increasingly prominent, leading to growing attention on the impact of meteorological factors on respiratory diseases. Research indicates that climate change can directly or indirectly impact the development and disease course of AR [12]. For example, by intensifying the symptoms of AR through altering the distribution, concentration, and human exposure patterns of allergenic substances including pollen grains and fungal conidia [13]. Recently, a growing number of studies have centered on the impact of meteorological factors on AR risk, but the findings have remained contentious due to conflicting results [14–17]. A study found that higher non-summer temperature was positively related to incidence of AR in Taiwan, China [16]. But Wang reported that higher temperature was related to less allergic rhinitis [14]. Likewise, a survey conducted by Silverberg, et al. reported that the relationship between relative humidity, precipitation and incidence of AR were statistically significant for every 1% rise in relative humidity and 10 mm rise in precipitation [15]. However, a large-scale multicenter study revealed no association between temperature, precipitation and AR risk [18]. Additionally, Hu el at. indicated that reduced relative humidity levels, diminished wind speed, and increased mean atmospheric pressure were linked to elevated clinical consultation rates for childhood AR [19].

The majority of systematic reviews and meta-analyses conducted to date have furnished evidence regarding the relationships between environmental factors and AR, predominantly centering on the association between airborne pollutants and AR [10, 20]. However, only one meta-analysis evaluated the association between temperature as a meteorological factor and AR [21]. Nonetheless, this meta-analysis focused exclusively on mean temperature, overlooking other clinically relevant temperature metrics such as maximum and minimum temperatures. Indeed, meteorological factors including humidity and precipitation also exert a critical role in AR. Additionally, amid the rapid accumulation of relevant primary research, there remains a paucity of comprehensive quantitative synthesis regarding the correlations between meteorological variables and AR. Given the growing impacts of climate change on environmental conditions and population health, there is an urgent need to integrate existing evidence to provide robust scientific support for the prevention and management of AR.

Against this backdrop, this study seeks to conduct a systematic collation of epidemiological evidence, quantitatively examine the strength and direction of associations between five meteorological factors (temperature, humidity, precipitation, wind speed, and atmospheric pressure) and AR, with the aim of addressing the key lacuna in current literature regarding the comprehensive assessment of multiple meteorological factors and further advancing the scientific understanding of the environmental triggers of AR.

## Methods

This systematic review and meta-analysis adhered to the Preferred Reporting Items for Systematic Reviews and Meta-Analyses (PRISMA) 2020 guideline [22] and has been registered in the International Prospective Register of Systematic Reviews (PROSPERO; ID: CRD42025637469). Furthermore, the study findings were presented in strict adherence to the methodological specifications outlined in the PRISMA 2020 checklist, which is documented in Supplementary Material 1.

### Search strategy

Guided by the design rationale of the PICO framework, we developed a targeted search strategy and conducted a systematic, involving an extensive retrieval of five major academic databases—PubMed, Scopus, Embase, Web of Science, and ProQuest—to identify eligible studies published up to December 1, 2025, with no restrictions imposed on language. The retrieval strategies for each database are available in Table S1. Moreover, this study conducted a manual review of the references cited in the selected articles and pertinent reviews to identify additional relevant literature.

### Inclusion and exclusion criteria

Studies meeting the eligibility criteria were included based on the following conditions: (I) The study design was observational studies, including cross-sectional, time series, case-control, cohort, ecological and case crossover studies. (II) the exposure factors were mean/maximum/minimum ambient temperature, relative humidity, precipitation, atmospheric pressure, and wind speed. (III) Various outcomes were documented through self-reports or guardian reports in questionnaires or interviews, reports of changes in AR symptoms, hospital records, and other sources. (IV) The outcomes concerning the influence of meteorological factors on AR are provided, including the odds ratios (ORs), relative risks (RRs), or regression coefficients (beta) with the corresponding 95% confidence interval (CIs). Furthermore, terms including hay fever, pollinosis, and pollen allergy, as referenced in the included studies, were classified as AR [21].

Studies meeting any of the subsequent exclusion criteria were excluded: (I) studies failing to report associations between meteorological factors and AR risk; (II) studies with incomplete data; (III) not provide original full data; (IV) research of animal studies, case report, commentaries, conference abstracts, letters and reviews.

### Literatures screening and data extraction

Retrieved references were uploaded to EndNote X9 software, with duplicate entries removed. Subsequently, eligible articles were reviewed on the titles, abstracts, and full-text. Two researchers (Q.L and W.G) independently extracted pertinent characteristics and study-specific information from the included literature using a pre-designed uniform data collection template. This process was followed by cross-checking to ensure consistency, and potential inconsistencies were addressed via deliberation in consultation with a third researcher (S.L) until a unified agreement was achieved. Subsequently, relevant information and characteristics were retrieved from eligible studies meeting the inclusion criteria, including the first author’s name, publication year, geographical region, type of study design, participant age range, and total sample size. Subsequently, relevant information and characteristics were retrieved from eligible studies meeting the inclusion criteria, including the first author’s name, publication year, geographical region, type of study design, participant age range, total sample size, meteorological data sources, exposure factors, AR outcomes, and adjustment for potential confounders, as well as effect estimates (OR/RR/beta) with 95% CIs.

### Quality assessment

The Newcastle-Ottawa-Scale (NOS) served as the instrument for assessing the quality of ecological, cross-sectional, and case-control studies [23]. The NOS conducts a comprehensive assessment of the study quality across three distinct dimensions, (I) selection (maximum five points); (II) comparability (maximum two points); and (III) outcome (maximum three points). A study was classified as high quality if the total points were seven or more. The quality evaluation instrument employed for time-series and case-crossover studies in this meta-analysis was modified based on prior investigations [24, 25]. The assessment items covered the verification of AR diagnosis (0 to 1 point), exposure measurements of meteorological factors (0 to 1 point), as well as control for potential confounders (0 to 3 points). If the diagnosis of AR is validated through the International Classification of Diseases (ICD) code or other globally recognized standardized criteria, a score of 1 is allocated; Conversely, a score of 0 is given if AR is defined solely by unvalidated self-reported or guardian-reported data if the study does not explicitly specify the diagnostic method for AR. The quality of exposure assessment is evaluated according to the measurement frequency and missing data (1 point was awarded if measurements were conducted daily with < 25% missing data, otherwise 0 point was allocated). Regarding the adjustment for confounders, a score ranging 1–3 was assigned if the analysis adjusted for long-term trends, seasonal variations, and air pollutants; otherwise, 0 points were allocated. Additionally, if the scores of the three evaluation items reach the maximum value, the study was categorized as high-quality. If one of the three items was scored 0 points, it indicated that the quality of study was low. According to different types of research designs, matched assessment tools were employed to appraise the quality of included references, which was classified into three levels: low, medium, and high quality [23, 24]. All studies neither meeting high-quality criteria nor classified as low-quality were judged medium quality. The above procedures were independently implemented by two researchers, and any discrepancies were addressed via deliberation in consultation with a third researcher.

### Risk of bias assessment

This study utilized the Risk Of Bias In Non-randomized Studies of Exposures (ROBINS-E) tool to appraise the methodological quality of studies included in the meta-analysis [26]. Tailored to assess the risk of bias (RoB) in exposure-outcome relationships among non-randomized studies, this instrument encompasses seven key bias domains: confounding, exposure measurement, selection bias, post-exposure intervention, outcome measurement, missing data, and selective reporting.

Every included study was evaluated independently by two research team members. The bias evaluation workflow proceeded as follows: After independently evaluating each bias domain, we comprehensively determined the overall RoB, anticipated direction of bias, and potential threats to conclusions. Per ROBINS-E’s default protocol, the overall RoB judgment is based on the domain with the highest risk. Each bias domain consists of multiple subdomains, with each subdomain assigned a three-level RoB rating (low, moderate, or high) during evaluation. The overall rating of each domain is derived from its subdomain ratings: a domain is classified as high RoB if any individual subdomain is assigned a high risk rating; low RoB if all subdomains are rated low; and moderate RoB if there is at least one moderate-RoB subdomain with no high-RoB subdomains.

### Statistical analysis

The effect size metrics employed to examine the association between meteorological factors and AR risk were ORs, RRs, or β coefficients, along with their 95% CIs. When reporting multiple model estimates, a multivariate adjusted model was utilized to contrast the effect estimates of the maximum exposure group against those of the minimum exposure group (or the reference group). When the outcome of interest is relatively common and effect sizes are small, we treated the RR as the OR in our analyses [27, 28]. The heterogeneity across included studies was assessed via the Cochran’s Q test and the Higgins *I²* statistic. The *P* value < 0.1 or I² > 50% was deemed to indicate substantial heterogeneity. When significant heterogeneity was present, a random-effect model was adopted to combine the effect sizes from separate studies [29, 30]. and subgroup analyses were conducted to explore the potential sources of the heterogeneity. The climate zone was classified using the Koppen climate classification [31]. If the study sites cover multiple climate zones, the study can be classified as a multiple climate zone study [21]. The country’s income levels were determined based on the World Bank’s classification of low-, middle- and high-income countries [32]. This study employed funnel plots and Egger’s test for the assessment of publication bias [33, 34]. Only if more than 5 studies were incorporated, funnel plots would be employed to gauge asymmetry [35]. The sensitivity analysis was performed by means of the leave-one-out method to assess the robustness of the aggregated estimates [36]. All statistical tests for the meta-analysis were performed using R version 4.4.2 statistical software. The difference was considered to be statistically significant when the two-sided *P* < 0.05.

### Evaluation of certainty of evidence

The study adopted the Grading of Recommendations Assessment, Development and Evaluation (GRADE) framework-a broadly acknowledged and employed instrument for appraising the certainty of cumulative evidence [37]. This framework was applied to methodically assess the certainty of evidence associating each exposure measure with the study outcome. GRADE categorizes evidence certainty into four distinct levels: very low, low, moderate, and high. The appraisal process involves a comprehensive evaluation of five downgrading elements and three upgrading elements: the downgrading standards encompass risk of bias, inconsistency, indirectness, imprecision, and publication bias, whereas the upgrading standards comprise a substantial effect size, the presence of a dose-response relationship, and plausible confounding that operates in the opposite direction. Following the assignment of quantitative scores to each assessment domain, we determined the final level of evidence certainty regarding the relationship between each exposure metric and the study outcome.

## Results

### Study selection and characteristics

**Fig. 1:**
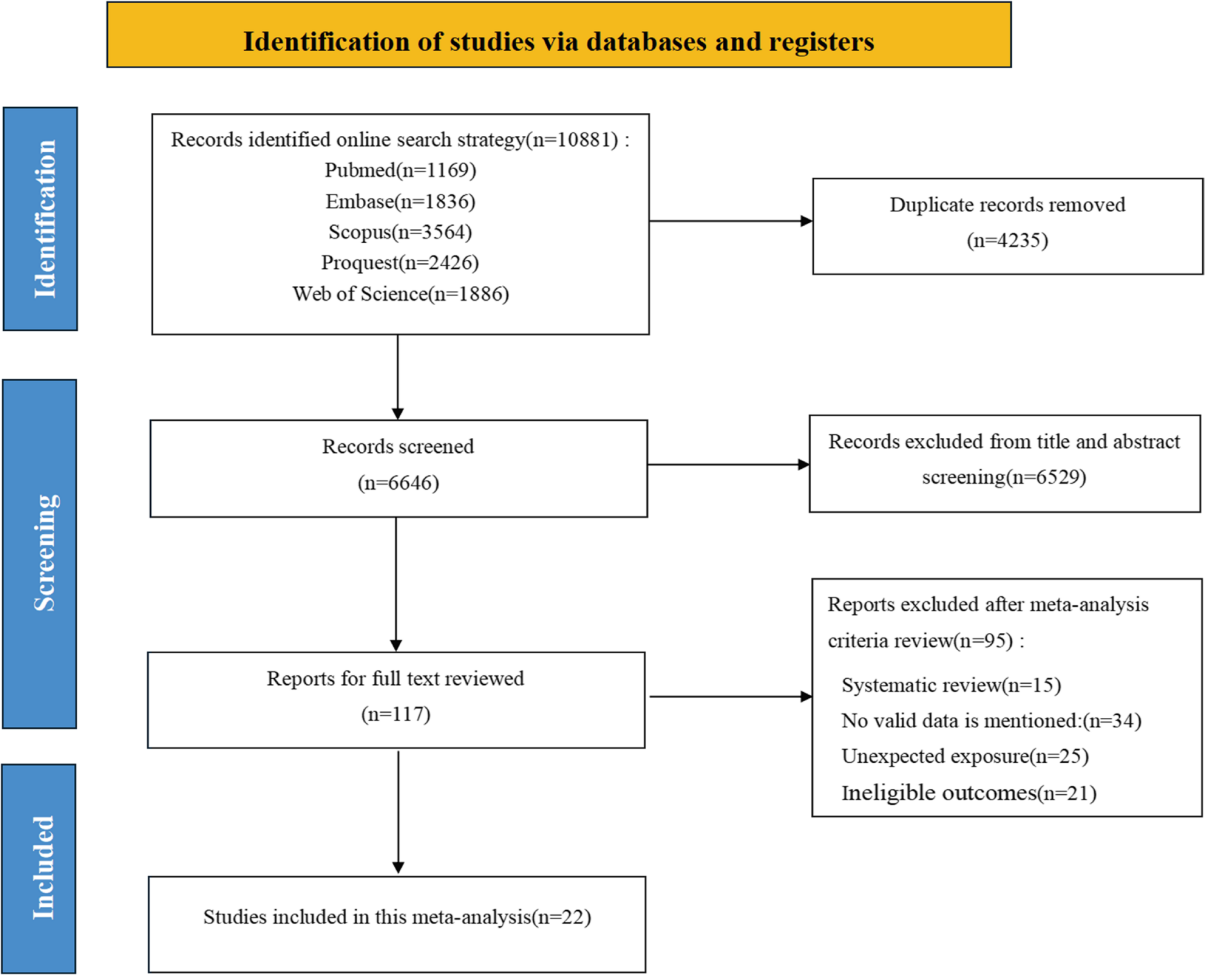
Flow diagram of the search strategy and study selection

The study selection workflow is illustrated in the PRISMA flow diagram **(**Fig. 1**)**. A total of 10,881 relevant articles were retrieved from the databases. Following the removal of duplicate entries and title-abstract screening, 117 articles were subjected to full-text evaluation, and 95 articles were excluded, the reasons were as follows: no valid data was mentioned in 34 articles, unexpected exposure occurred in 25 articles, 15 articles were systematic reviews, and 21 articles had ineligible outcomes. Finally, 22 studies were included in this meta-analysis.

Table 1 presents an overview of the characteristics of all included eligible studies. Regarding the exposure factors, there are 21 studies for temperature, 12 studies for relative humidity, 6 studies for precipitation, 4 studies for atmospheric pressure, 3 studies for wind speed. All included study locations spanned widely, covering Asia, Europe, Oceania, and America, and comprised a combined sample size of 5,218,796 participants. They included 11 cross-sectional studies, 7 time-series studies, 2 ecological studies, 1 crossover-case study and 1 case-control study. All studies were assessed as medium or high quality, with 12 classified as high quality and 10 as medium quality. The details of quality assessment can be found in Tables S2–S4.

**Table 1:** Characteristics of included studies

Study	Location	Study design	Age	Sample size	Exposure source	Exposure factors	Climate zone	Income group	Outcome Assessment	Outcome Type	Quality score
Lee et al. 2003 [16]	Taiwan, China	CS	< 18	312,873	Environmental Protection Administration monitoring stations	MT; RH	Subtropical	Middle	ISAAC Questionnaire	Self-reported	9/10
Hughes et al., 2011 [38]	Australia	CCS	≤ 14	527	Australian National Climate Centre of the Bureau of Meteorology Research Centre	MT; RH	Multiple	High	Standardized questionnaire and a face-to-face interview	Self-reported	8/9
Hsieh et al. 2020 [39]	Taiwan, China	CC	0–18 19–49 ≥ 50	140,365	Environmental monitoring stations in Taiwan	MT; RH	Subtropical	Middle	ICD-9 CM	Medical records	4/5
Hu et al. 2020 [19]	Shanghai, China	TS	< 18	2,410,392	Shanghai Meteorological Center	MT; RH; P; WS	Subtropical	Middle	ICD-10, J30	Medical records	5/5
Duan et al. 2019 [40]	Hefei, China	TS	< 18	37,221	Hefei Meteorology Bureau	RH	Subtropical	Middle	ICD-10, J30	Medical records	3/5
Weiland et al. 2004 [41]	Western Europe (12 countries)	ES	6–7 13–14	463,801	Measurement stations’ data from World Weather Guide	MT; RH	Multiple	High	ISAAC core & video questionnaires	Self-reported	7/10
Zanolin et al. 2004 [42]	Italia	ES	20–44	18,873	Institute of Atmospheric and Oceanic Sciences and Regional Agencies for the Protection of the Environment	MT	Multiple	High	Standardized questionnaire	Self-reported	7/10
Wang et al. 2021 [14]	China	CS	> 18	40,279	The website of National Bureau of Statistics	MT	Multiple	Middle	CCHH questionnaires	Self-reported	6/10
Wang et al. 2018 [17]	Inner Mongolia, China	CS	0–17 18–59 ≥ 60	6,043	The China Meteorological Data Sharing Service Network	MT; RH; P; AP; WS	Temperate	Middle	ARIA document	Medical records	8/10
Qiu et al. 2022 [43]	Chongqing, China	CS	10–13	4,146	Weather Underground	MT; RH	Subtropical	Middle	ISAAC Questionnaire; Nasal symptoms report	Medical records	6/10
Kurt et al. 2007 [44]	Turkey	CS	6–15	25,843	GMA, MOEF, GSI, and regional local meteorological offices	MT; RH; AP	Multiple	Middle	Standardized questionnaire	Self-reported	6/10
Wang et al. 2019 [18]	China	CS	≥ 18	40,279	Weather Underground	MT; P	Multiple	Middle	CCHH questionnaires	Self-reported	6/10
Silverberg et al. 2015 [15]	USA	CS	0–17	91,642	National Climatic Data Center	MT; RH; P	Multiple	High	NSCH question	Self-reported	7/10
Gao et al. 2021 [45]	Xinxiang, China	TS	< 15 15–64 ≥ 65	14,965	China Meteorological Data Sharing Service System	MT	Subtropical	Middle	ICD-10, J30	Medical records	4/5
Breton et al. 2006 [46]	Montreal, Canada	TS	0–19 19–59 ≥ 60	7,667	The Montreal/Pierre Elliott Trudeau weather station	MaxT; P	Temperate	High	ICD-9-CM	Medical records	3/5
Todkill et al. 2020 [47]	London, England	TS	NA	186,401	The UK’ s national weather service	MT; P	Temperate	High	UK Read Coding System	Medical records	5/5
Bhattacharyya et al. 2009 [48]	USA	CS	≥ 18	851,581	The National Climatic Data Center	MT	Multiple	High	National Health Interview Survey	Self-reported	8/10
Kim et al. 2011 [49]	Seoul, Korea	TS	≥ 18	4,715	The Korea Meteorological Administration	MinT	Temperate	High	Skin prick tests	Medical records	3/5
Upperman et al. 2017 [50]	USA	CS	≥ 18	505,386	National Climatic Data Center	MaxT	Multiple	High	National Health Interview Survey	Self-reported	8/10
He et al. 2017 [51]	Shanghai, China	CS	3–16	351	The Shanghai Meteorological Service and Shanghai Environmental Protection Bureau	MT	Subtropical	Middle	ICD-10, J30	Self-reported	7/10
Niu et al. 2025 [52]	China	CS	3–6	400,13	ChinaHighAirPollutants (CHAP) dataset	MT	Multiple	Middle	ISAAC Questionnaire	Self-reported	9/10
Wang et al. 2025 [53]	Changchun, China	TS	< 15 15–64 ≥ 65	15,338	China Meteorological Data Service Centre	MT	Temperate	Middle	ICD-10, J30	Medical records	4/5

*TS* Time-series study, *CS* Cross-sectional study, *CC* Case-crossover study, *CCS* Case-control study, *ES* Ecological study, *NA* Not available, *UN* Unknown, *MT* Mean Temperature (^0^C), *RH* Relative humidity (%), *AP* Atmospheric pressure (hPa), *P* Precipitation (mm), *WS* Wind speed (m/s), *MaxT* Maximum temperature, *MinT* Minimum temperature, *GMA* General Meteorology Agency, *MOFF* Ministry of Environment and Forests, *GSI* Government Statistical Institute, *ICD-10* International Classification of Disease, 10th version, *ICD-9 CM* International Classification of Diseases, the 9th Revision, Clinical Modification, *ISAAC* International Study of Asthma and Allergies in Childhood, *CCHH* China, Children, Homes, Health, *ARIA* Allergic Rhinitis and Its Impact on Asthma

### Overall effects and subgroup analyses

**Fig. 2:**
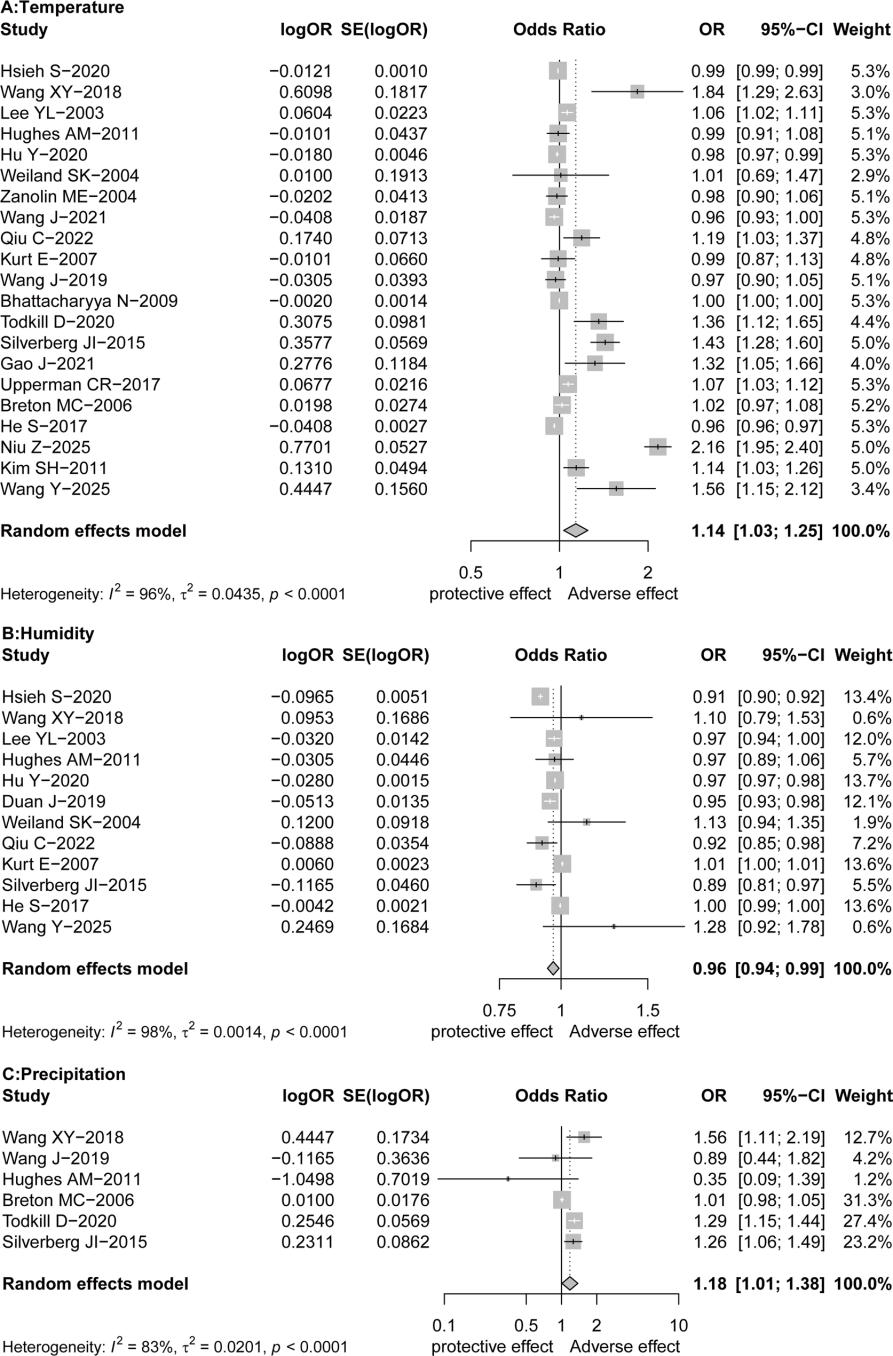
Forest plot of the relationship between meteorological factors and allergic rhinitis. **A** Temperature; **B** Relative humidity; **C** Precipitation

The effect sizes are depicted in the forest plots (Fig. 2 and Fig. S1). The overall pooled results showed that a 1 °C increase in temperature is associated with a 14% heightened risk of AR [OR (95%CI) = 1.14 (1.03–1.25)]. Meanwhile, relative humidity has a significant association with AR, for per 1% increase in relative humidity, the risk of AR decreases by 4% [OR (95%CI) = 0.96 (0.94–0.99)]. Moreover, precipitation was found to be statistically significantly associated with AR [OR (95%CI) = 1.18 (1.01–1.38)]. However, atmospheric pressure [OR (95%CI) = 0.85 (0.50–1.46)] and wind speed [OR (95%CI) = 0.76 (0.56–1.04)] were not significantly associated with AR. Besides, significant heterogeneity was observed among the studies, it was essential to conduct subgroup analyses. Subgroup analyses were performed to investigate potential sources of variability, categorized by temperature measure, climate zone, country’s income level, literature quality and outcome type (Table 2), The key findings are as follows.

**Table 2:** Subgroup analysis of meteorological factors and AR risk

Meteorological Variables	Subgroup Types	*N*	Heterogeneity		OR (95%CI)
			I² (%)	*P*	
Temperature	**Temperature measure**				
	Mean Temperature	18	97	< 0.0001	**1.15(1.03–1.29)**
	Maximum Temperature	2	47	0.1700	**1.05(1.00–1.10)**
	Minimum Temperature	1	NA	NA	**1.14(1.03–1.26)**
	**Climate Zone**				
	Subtropical	6	96	< 0.0001	1.03(0.97–1.10)
	Temperate	5	81	0.0003	**1.27(1.03–1.56)**
	Multiple	10	97	< 0.0001	1.12(0.95–1.32)
	**Country’s Income**				
	Middle- Income	12	97	< 0.0001	**1.18(1.01–1.37)**
	High- Income	9	88	< 0.0001	1.09(0.99–1.20)
	**Literature quality**				
	Medium-Quality	9	76	< 0.0001	1.06(0.99–1.14)
	High-Quality	12	98	< 0.0001	**1.18(1.01–1.37)**
	**Outcome Type**				
	Medical Records	9	85	< 0.0001	**1.18(1.05–1.33)**
	Self-Reported	12	98	< 0.0001	1.10(0.96–1.26)
Humidity	**Climate Zone**				
	Subtropical	6	98	< 0.0001	**0.95(0.93–0.98)**
	Temperate	2	0	0.5248	1.19(0.94–1.50)
	Multiple	4	68	0.0257	0.98(0.91–1.05)
	**Country’s Income**				
	Middle-Income	9	98	< 0.0001	**0.97(0.94–0.99)**
	High-Income	3	65	0.0575	0.97(0.86–1.09)
	**Literature quality**				
	Medium-Quality	5	99	< 0.0001	0.95(0.91–1.01)
	High-Quality	7	94	< 0.0001	**0.98(0.96–0.99)**
	**Outcome Type**				
	Medical Records	6	97	< 0.0001	**0.94(0.91–0.98)**
	Self-Reported	6	80	0.0002	0.99(0.96–1.01)
Precipitation	**Climate Zone**				
	Temperate	3	91	< 0.0001	1.22(0.96–1.54)
	Multiple	3	51	0.1317	0.99(0.60–1.64**)**
	**National Income**				
	Middle	2	48	0.1635	1.29(0.77–2.17)
	High	4	88	< 0.0001	1.15(0.97–1.35)
	**Literature Quality**				
	Medium-Quality	2	0	0.7282	1.01(0.98–1.05)
	High-Quality	4	37	0.1927	**1.29(1.18–1.41)**
	**Outcome Type**				
	Medical Records	3	91	< 0.0001	1.22(0.96–1.54)
	Self-Reported	3	51	0.1317	0.99(0.60–1.64)

Statistically significant results are in bold

*N* the number estimates, *NA* Not available, *OR* Odds ratio, *CI* Confidence interval

### Temperature measure

In the subgroup analysis of temperature measures, mean temperature (*n* = 18), maximum temperature (*n* = 2) and minimum temperature (*n* = 1) were all positively correlated with an increased risk of AR [OR (95%CI) = 1.15 (1.03–1.29) for mean temperature, OR (95%CI) = 1.05 (1.00–1.10) for maximum temperature, OR (95%CI) = 1.14 (1.03–1.26) for minimum temperature]. The subgroup of maximum temperature demonstrated reduced heterogeneity, and *I*^2^ was 47% (*P* = 0.17). The detailed forest plot is shown in Fig. S2.

### Climate zone

In the climate zone-stratified subgroup analysis of study locations, ambient temperature in temperate zone (*n* = 5) demonstrated a significant association with AR [OR (95%CI) = 1.27 (1.03–1.56)]. However, studies in subtropical zone (*n* = 6) and multiple climate zone (*n* = 10) showed no significant difference between ambient temperature and AR [OR (95%CI) = 1.03 (0.97–1.10) for subtropical zone, OR (95%CI) = 1.12 (0.95–1.32) for multiple climate zone]. The studies of subtropical zone (*n* = 6) showed significant association with AR and humidity [OR (95%CI) = 0.95 (0.93–0.98)]. Nevertheless, studies in multiple climate zone (*n* = 4) and temperate zone (*n* = 2) indicated humidity wasn’t significantly associated with AR [OR (95%CI) = 0.98 (0.91–1.05) for multiple climate zone, OR (95%CI) = 1.19 (0.94–1.50) for temperate zone]. For precipitation, studies in the temperate zone (*n* = 3) and the multiple climate zones (*n* = 3) have found that no significant association was observed between precipitation and AR [(OR (95%CI) = 0.99 (0.60–1.64) for multiple climate zones, OR (95%CI) = 1.22 (0.96–1.54) for the temperate zone)]. The detailed forest plot is shown in Figs. S2–S4.

### Income level

In the subgroup analysis stratified by countries’ income level, a statistically significant association between temperature and AR risk was demonstrated in middle-income countries [OR (95%CI) = 1.18 (1.01–1.37)], in contrast to high-income countries (*n* = 9) which showed no significant correlation [OR (95%CI) = 1.09 (0.99–1.20)]. Additionally, nor was any significant association found between precipitation and AR risk in the middle-income countries (*n* = 2) and high-income countries (*n* = 4) [OR (95%CI) = 1.29 (0.77–2.17) for middle-income countries, OR (95%CI) = 1.15 (0.97–1.35) for high-income countries]. When conducting subgroup analysis of humidity stratified by countries’ income level, the studies of middle-income countries (*n* = 9) showed the risk of AR associated with 1% increase in relative humidity were decreased by 3% [OR (95%CI) = 0.97 (0.94–0.99)], and this relationship is statistically significant. However, in high income countries (*n* = 3), no significant association was identified between relative humidity and AR [OR (95%CI) = 0.97 (0.86–1.09)]. The detailed forest plot is shown in Figs. S2–S4.

### Literature quality

Stratification by literature quality identified uniform results for the associations between the three meteorological factors and AR risk: significant associations were uniformly reported in high-quality studies, whereas no significant associations were observed in medium-quality studies. Specifically, high-quality evidence (*n* = 12) demonstrated that a 1 °C rise increased AR risk by 18% [OR (95%CI) = 1.18 (1.01–1.37)]. In contrast, in medium-quality studies (*n* = 9), no significant relationship between temperature and AR was found [OR (95%CI) = 1.06 (0.99–1.14)]. Furthermore, high-quality (*n* = 7) studies found a significant association between relative humidity and AR risk, with medium-quality studies (*n* = 5) showing no statistical significance [OR (95%CI) = 0.95 (0.91–1.01) for medium-quality; OR (95%CI) = 0.98 (0.96–0.99) for high-quality]. For precipitation, high-quality (*n* = 4) studies identified a statistically robust correlation between precipitation and AR risk [OR (95%CI) = 1.29 (1.18–1.41)], and indicated less heterogeneity (*I*^*2*^ decreased to 37%, *P* = 0.1927). by contrast, no significant correlation was found between precipitation and the risk of AR in studies of medium quality [OR (95%CI) = 1.01 (0.98–1.05)]. Comprehensive forest plots are provided in Figs. S2–S4.

### Outcome type

In the subgroup analysis of Outcome type, 9 studies using medical record data confirmed a significantly positive statistical association between temperature exposure and AR risk [OR (95% CI) = 1.18 (1.05–1.33)]. Conversely, 12 studies based on self-reported outcomes failed to identify such a significant association [OR (95%CI) = 1.10 (0.96–1.26)]. Regarding relative humidity, five studies utilizing medical record data demonstrated a statistically significant inverse association with AR [OR (95%CI) = 0.94 (0.91–0.98)]. On the other hand, six studies based on self-reported outcomes failed to detect a similarly significant relationship [OR (95%CI) = 0.99 (0.96–1.01)]. With respect to precipitation, studies utilizing medical records (*n* = 3) failed to identify a statistically significant association with AR risk [OR (95%CI) = 1.22 (0.96–1.54)], and the same was true for those using self-reported outcomes (*n* = 3) [OR (95%CI) = 0.99 (0.60–1.64)]. The detailed forest plot is shown in Figs. S2–S4.

### Sensitivity analysis and publication bias

Results of the sensitivity analysis indicated that the relationships between temperature, relative humidity, and AR exhibited general stability. However, the association between precipitation and AR risk became insignificant after omitting Wang XY et al. study [17] [OR (95%CI) = 1.13 (0.97–1.33)], Todkill D et al. study [47] [OR (95%CI) = 1.14 (0.93–1.41)] or Silverberg JI et al. study [15] [OR (95%CI) = 1.15 (0.93–1.43)]. Specific particulars can be found in Fig**. **S5**.** For publication bias assessment, Egger’s test results showed that the P-value for the temperature-related analysis was 0.067, that for the humidity-related analysis was 0.625, and that for the precipitation-related analysis was 0.386. Accordingly, after performing funnel plot assessment and Egger’s test (with *P* > 0.05), no signs of publication bias were identified. The funnel plots associated with these analyses are provided in Figs. S6–S8.

### Risk of bias (RoB) assessment

**Fig. 3:**
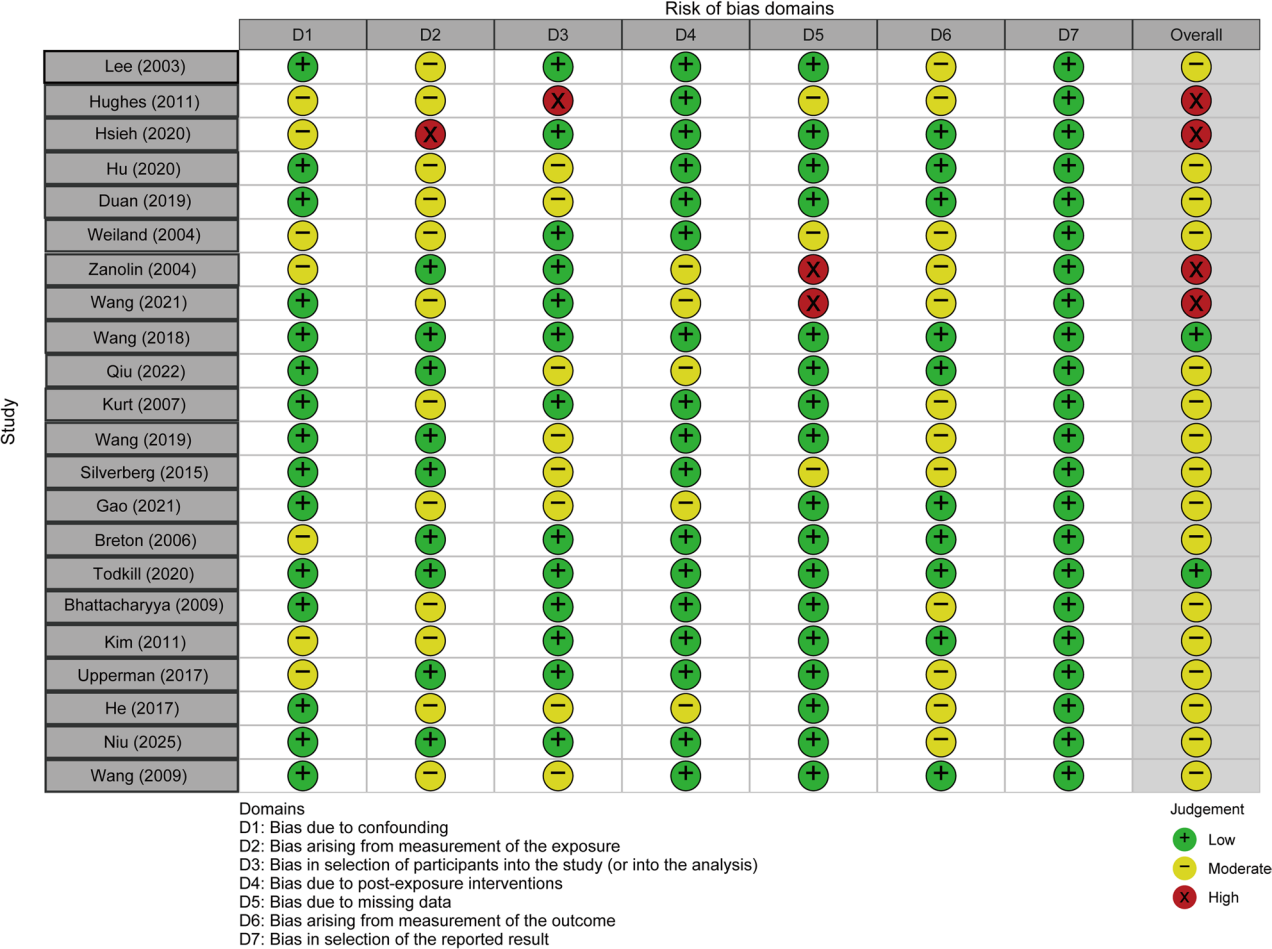
Summary of the risk of bias assessment for all included studies in the meta-analysis

Figure 3 depicts the results of the risk of bias assessment for the included studies: Among the included studies, two were classified as having a low risk of bias, 16 as having a moderate risk of bias, and 4 as having a high risk of bias, with the main sources of bias being exposure measurement and outcome measurement.

### Certainty of evidence

For the assessment of global certainty of evidence related to exposure variables in all included studies, we adopted the GRADE (Grading of Recommendations Assessment, Development and Evaluation) methodology. Results indicated that temperature and humidity were assigned a high evidence quality rating, precipitation a low rating, and other indicators (such as wind speed and atmospheric pressure) a very low rating. Detailed information can be found in **Table **S5**.**

## Discussion

This study synthesized evidence from 22 observational studies to elucidate the relationship between meteorological factors and AR risk. We found temperature and precipitation were positively associated with AR risk, while relative humidity exhibited a protective effect. However, no significant correlation was found between wind speed, atmospheric pressure and AR.

### The relationship between temperature and AR

The present meta-analysis showed that exposure to elevated air temperatures exhibited a significant association with an increased risk of AR. Mechanistically, rising temperature may induce the pollen season to start earlier and prolong the flowering period, thus increasing the duration and quantity of human exposure to pollen [54–56]. For example, research conducted in North America indicates that during the past 30 years, owing to the upward trend in global temperature and CO₂ levels, the pollen season has prolonged by an average of 20 days, and pollen concentrations have increased by 21% [57]. While pollen serves as a key allergen driving AR risk in regions like North America, region-specific allergen profiles may shape distinct temperature-related AR pathogenesis, a consideration of notable relevance, given that most of the studies included in the present meta-analysis were performed in Asian nations. Existing epidemiological evidence clearly indicates that house dust mites (HDMs) represent the primary pathogenic trigger for AR in Asian populations [58, 59]. Accumulating evidence demonstrates [52, 60] that abrupt surges in ambient temperature can substantially accelerate the proliferation of HDMs. Cumulative exposure to HDM allergens impairs the structural integrity of the respiratory epithelial barrier, thereby eliciting local allergic inflammatory responses in the respiratory tract [61]. Furthermore, persistent high-temperature conditions tend to exacerbate the intensity of extreme meteorological events such as rainstorms and floods [60]. These events, in turn, generate hot and humid microenvironments that are highly conducive to HDM survival and dispersion. The hot and humid microenvironments thus generated not only facilitate the reproduction and dispersion of HDMs but also further compromise the functional integrity of the respiratory mucosal barrier, inducing chronic airway inflammation [62]. This cascade of effects ultimately contributes to the indirect exacerbation of clinical symptom severity in AR patients.

Beyond the impact on pollen and HDM, high temperatures also directly affect the human respiratory system. Existing studies indicate that extremely high temperatures can cause significant harm to the respiratory system [63]. On one hand, elevated temperatures directly compromise the structural integrity of the respiratory epithelial barrier. On the other hand, high temperature exerts an effect on the body’s thermoregulatory system, leading to increased tidal volume and respiratory frequency [60, 64]. Subsequently, activation of bronchopulmonary vagal nerve fibers and upregulated expression of transient receptor potential vanilloid 1 (TRPV1) and transient receptor potential vanilloid 4 (TRPV4) induce reflex bronchoconstriction and heightened specific airway resistance [65, 66]. Additionally, heat stress induces the expression of heat shock proteins, thereby impairing epithelial barrier function and provoking airway inflammation, which ultimately elevates AR incidence [52, 60].

Notably, in the subgroup analysis stratified by climate zones, temperature exhibited a statistically robust positive association with AR risk in temperate regions. Notably, in the subgroup analysis stratified by climate zones, temperature exhibited a statistically robust positive association with AR risk in temperate zone. This might be because temperate zone has distinct four seasons and relatively obvious temperature changes. When exposed to temperature fluctuations beyond the body’s adaptability, the physiological function of the nasal mucosa may be affected, making it easier for allergens to invade the human body, which may give rise to increased incidence of allergic conditions like AR [67, 68]. Within the subgroup analysis of temperature indicators, mean temperature was significantly and positively correlated with AR risk, exhibiting a stronger association than maximum temperature. Although maximum temperature reflects the body’s acute stress response to short-term high temperatures, its impact is confined to specific heatwave event [63]. In contrast, mean temperature encompasses long-term climate trends, including the CO₂-induced enhancement of pollen allergenicity and exacerbation of regional heat island effects [54, 69]. Furthermore, the effect of temperature on AR were most pronounced in high-quality studies, while medium-quality studies did not find a significant association. This phenomenon might be attributable to high-quality studies featuring more rigorous research designs and more effective control of confounding variables [70], thus enabling a more accurate analysis of the true association between temperature and AR risk. In the correlation analysis of temperature and AR, outcomes based on medical records show a stronger statistical correlation, while self-reported outcomes do not reveal a significant association. This difference is mainly rooted in the greater objectivity and accuracy exhibited by medical records—AR cases are defined through standardized clinical diagnostic criteria, and uniform medical assessment standards are followed, thus guaranteeing the dependability of AR-related outcomes. On the other hand, self-reported outcomes are prone to the effects of subjective judgment and recall bias. Additionally, there are variations in AR symptom reporting thresholds among different population groups [71]. These factors collectively lead to inconsistencies between the evaluated and actual AR prevalence, thereby masking the genuine correlation between meteorological factors and AR.

### The relationship between humidity and AR

The results of the present study indicate that ambient humidity plays a protective role in mitigating AR risk, primarily through its influence on pollen dispersion and respiratory epithelial function. A comprehensive review indicates that under low humidity conditions, pollen release is enhanced, facilitating its dispersion and transportation in the atmosphere [72, 73]. Conversely, elevated humidity correlates with a decrease in airborne pollen concentrations, as pollen grains absorb moisture, increasing their weight and limiting their ability to remain airborne [74, 75]. In addition to its effects on pollen dynamics, inhaled air humidity significantly impacts respiratory epithelial integrity and mucociliary clearance. Low humidity levels dehydrate the nasal epithelium, impairing ciliary motility and compromising the mucosal barrier, thereby elevating AR risk [76] On the other hand, while moderate humidity supports epithelial hydration and efficient allergen clearance, persistently high humidity creates an environment conducive to the proliferation of molds and dust mites, which can exacerbate inflammation and worsen AR symptoms [77]. It is noteworthy that the majority of AR cases included in this study were pollen-induced, which aligns well with the proposed mechanisms.

Subgroup analyses further revealed that humidity exerts a more notable protective effect on AR in subtropical regions. This could be due to the high-temperature conditions in subtropical areas, which allow humidity to contribute to the preservation of the nasal mucosa’s integrity. Previous studies have shown that inhaling warm air that has undergone humidification treatment helps alleviate the symptoms of AR, improve nasal ventilation function, and reduce the nasal cavity’s sensitivity to allergen provocation [78]. When stratifying by national income level, this study identified a statistically robust correlation between humidity and AR risk in middle-income countries, as opposed to high-income countries where no significant association was observed. This discrepancy is likely due to the differing prevalence of indoor climate control technologies between income groups. In high-income countries, widespread use of air conditioners, dehumidifiers, and humidifiers maintains stable indoor humidity levels, decoupling outdoor humidity from human exposure and blunting its association with AR [79].

### The relationship between precipitation and AR

It was found in the present study that higher precipitation levels were linked to a heightened risk of AR. This observation aligns with previous studies indicating that heavy rainfall can trigger the release of allergenic substances including pollen and fungal spores, which may exacerbate AR symptoms [80]. For instance, D’Amato et al. have demonstrated that precipitation may cause pollen grains to break, thereby generating atmospheric biological aerosols that carry allergens and further contributing to the allergen burden in the environment [81].

However, sensitivity analysis indicated that the relationship between precipitation and AR risk lacks full robustness. This points to the observed association may be influenced by study-specific attributes, including regional climatic differences, population demographics, or methodological variations. For example, Wang XY et al. focused on a region with high pollen exposure in northern China, where precipitation patterns may have a more pronounced effect on pollen dispersion and allergen release [17]. Similarly, Silverberg JI et al. [15]. and Todkill D et al. [47]. examined populations in the United States and the United Kingdom, respectively, where climatic conditions and allergen profiles may differ from those in other regions.

### Strengths and limitations

The current study possesses notable advantages. For one thing, it is the first systematic review and meta-analysis to examine the relationships between various meteorological factors and AR. providing important epidemiological evidence. Moreover, the study not only provides statistical associations but also explores the potential biological mechanisms through temperature, humidity, and precipitation influencing AR. This research also has several inherent limitations that ought to be highlighted. has several limitations that need to be noted. Firstly, as the majority of the included studies adopt cross-sectional designs, it might restrict the conclusions that could be reached. Secondly, age and gender, which are potentially the sources of heterogeneity, made it impossible to perform subgroup analysis on account of the restricted quantity of the studies incorporated. Third, while subgroup analyses were performed to identify the sources of heterogeneity, substantial heterogeneity will inevitably impair the robustness of the ultimate findings. Forth, even though the included studies are sourced from multiple regions, the high proportion of studies originating from Asian countries could preclude a precise representation of how meteorological factors relate to AR on a global level. Furthermore, given that relevant studies on meteorological variables such as diurnal temperature range and sunshine duration are relatively scarce, these variables have not yet been incorporated into the analytical framework of this study. There is an urgent need to conduct more high-quality studies in the future to further enrich and improve the evidence base regarding the association between meteorological factors and AR.

## Conclusions

In conclusion, the findings provide epidemiological evidence that temperature and precipitation may increase the risk of AR, while humidity may reduce the risk of AR. Temperature-related risk is more notable with mean temperature or in temperate zones, humidity-related risk is more prominent in subtropical and middle-income areas, and the impacts of temperature and precipitation are more evident in high-quality studies. In the future, emphasis should be placed on considering the role of the interactions among various meteorological factors in relation to AR, so as to clarify the relationship and the relevant mechanisms.

## Supplementary Information

Supplementary Material 1. Completed PRISMA 2020 Checklist

Supplementary Material 2: Table S1 Search Strategies for Each Database. Table S2 Quality assessment of time-series analysis and case-crossover studies. Table S3 Quality assessment of ecological and cross-sectional studies using the Newcastle-Ottawa-Scale. Table S4 Quality assessment of case-control studies using the Newcastle-Ottawa-Scale. Table S5 GRADE certainty of evidence rating and rationale.

Supplementary Material 3: Fig. S1 Forest plot of the relationship between atmospheric pressure, wind speed and allergic rhinitis. A. Atmospheric pressure; B. Wind speed. Fig. S2 Subgroup analysis of allergic rhinitis and temperature. A. Temperature Measure; B. Climate Zone; C. Country’s Income Level; D. Literature-Quality; E. Outcome type. Fig. S3 Subgroup analysis of between allergic rhinitis and humidity. A. Climate Zone; B. Country’s Income Level; C. Literature-Quality; D. Outcome type. Fig. S4 Subgroup analysis of between allergic rhinitis and Precipitation. A. Climate Zone; B. Country’s Income Level; C. Literature-Quality; D. Outcome type. Fig. S5 Sensitivity analysis of meteorological factors and allergic rhinitis. A. Temperature; B. Humidity; C. Precipitation. Fig. S6 Temperature and allergic rhinitis funnel plot. Fig. S7 Humidity and allergic rhinitis funnel plot. Fig. S8 Precipitation and allergic rhinitis funnel plot.

## Data Availability

The raw data supporting the findings of this study will be made publicly available by the research team, without any undue restrictions.

## Abbreviations

AR: Allergic Rhinitis
IgE: Immunoglobulin E
PRISMA: Preferred Reporting Items for Systematic Reviews and Meta-Analyses
ORs: Odds Ratios
RRs: Relative Risks
HR: Hazard Ratios
CIs: Confidence Intervals
NOS: Newcastle-Ottawa-Scale
ICD: International Classification of Diseases
TRPV1: Transient Receptor Potential Vanilloid 1
TRPV4: Transient Receptor Potential Vanilloid 4

## References

[1] Sun W, Ding C, Jiang Z, et al. The impact of ambient air pollution on allergic rhinitis symptoms: a prospective follow-up study. Toxics. 2024;12(9):663. 39330591 10.3390/toxics12090663 PMC11436010

[2] Zhang Y, Yan B, Zhu Z, et al. Efficacy and safety of Stapokibart (CM310) in uncontrolled seasonal allergic rhinitis (MERAK): an investigator-initiated, placebo-controlled, randomised, double-blind, phase 2 trial. EClinicalMedicine. 2024;69:102467. 38356731 10.1016/j.eclinm.2024.102467 PMC10864214

[3] Canonica GW, Bousquet J, Mullol J, et al. A survey of the burden of allergic rhinitis in Europe. Allergy. 2007;62(Suppl 85):17–25. 17927674 10.1111/j.1398-9995.2007.01549.x

[4] Meltzer EO, Farrar JR, Sennett C. Findings from an online survey assessing the burden and management of seasonal allergic rhinoconjunctivitis in US patients. J Allergy Clin Immunol Pract. 2017;5(3):779–e789776. 27914815 10.1016/j.jaip.2016.10.010

[5] Katelaris CH, Lee BW, Potter PC, et al. Prevalence and diversity of allergic rhinitis in regions of the world beyond Europe and North America. Clin Exp Allergy. 2012;42(2):186–207. 22092947 10.1111/j.1365-2222.2011.03891.x

[6] Brożek JL, Bousquet J, Agache I, et al. Allergic rhinitis and its impact on asthma (ARIA) guidelines-2016 revision. J Allergy Clin Immunol. 2017;140(4):950–8. 28602936 10.1016/j.jaci.2017.03.050

[7] Blaiss MS, Hammerby E, Robinson S, et al. The burden of allergic rhinitis and allergic rhinoconjunctivitis on adolescents: A literature review. Ann Allergy Asthma Immunol. 2018;121(1):43–e5243. 29626629 10.1016/j.anai.2018.03.028

[8] Sapsaprang S, Setabutr D, Kulalert P, et al. Evaluating the impact of allergic rhinitis on quality of life among Thai students. Int Forum Allergy Rhinol. 2015;5(9):801–7. 25899701 10.1002/alr.21540

[9] Meng Y, Wang CZL. Advances and novel developments in allergic rhinitis. Allergy. 2020;75(12):3069–76. 32901931 10.1111/all.14586

[10] Li S, Wu W, Wang G, et al. Association between exposure to air pollution and risk of allergic rhinitis: a systematic review and meta-analysis. Environ Res. 2022;205:112472. 34863689 10.1016/j.envres.2021.112472

[11] Lisik D, Ermis S, Ioannidou A, et al. Siblings and risk of allergic rhinitis: a systematic review and meta-analysis. Pediatr Allergy Immunol. 2023;34(7):e13991. 37492922 10.1111/pai.13991

[12] D’Amato G, Holgate ST, Pawankar R, et al. Meteorological conditions, climate change, new emerging factors, and asthma and related allergic disorders. World Allergy Organ J. 2015;8(1):25. 26207160 10.1186/s40413-015-0073-0 PMC4499913

[13] Barnes CS. Impact of climate change on pollen and respiratory disease. Curr Allergy Asthma Rep. 2018;18(11):59. 30238321 10.1007/s11882-018-0813-7

[14] Wang J, Zhang Y, Li B, et al. Asthma and allergic rhinitis among young parents in China in relation to outdoor air pollution, climate and home environment. Sci Total Environ. 2021;751:141734. 32882555 10.1016/j.scitotenv.2020.141734

[15] Silverberg JI, Braunstein M, Lee-Wong M. Association between climate factors, pollen counts, and childhood hay fever prevalence in the United States. J Allergy Clin Immunol. 2015;135(2):463–9. 25304658 10.1016/j.jaci.2014.08.003

[16] Lee YL, Shaw CK, Su HJ, et al. Climate, traffic-related air pollutants and allergic rhinitis prevalence in middle-school children in Taiwan. Eur Respir J. 2003;21(6):964–70. 12797489 10.1183/09031936.03.00094602

[17] Wang XY, Ma TT, Wang XY, et al. Prevalence of pollen-induced allergic rhinitis with high pollen exposure in grasslands of Northern China. Allergy. 2018;73(6):1232–43. 29322523 10.1111/all.13388 PMC6033040

[18] Wang J, Zhao Z, Zhang Y, et al. Asthma, allergic rhinitis and eczema among parents of preschool children in relation to climate, and dampness and mold in dwellings in China. Environ Int. 2019;130:104910. 31226554 10.1016/j.envint.2019.104910

[19] Hu Y, Xu Z, Jiang F, et al. Relative impact of meteorological factors and air pollutants on childhood allergic diseases in Shanghai, China. Sci Total Environ. 2020;706:135975. 31841850 10.1016/j.scitotenv.2019.135975

[20] Zhang ZQ, Li JY, Guo Q, et al. Association between air pollution and allergic upper respiratory diseases: a meta-analysis. Eur Respir Rev. 2025. 10.1183/16000617.0266-2024. 40562438 10.1183/16000617.0266-2024 PMC12220745

[21] Wei Rong CW, Salleh H, Nishio H, et al. The impact of increasing ambient temperature on allergic rhinitis: a systematic review and meta-analysis of observational studies. Sci Total Environ. 2024;947:174348. 38960184 10.1016/j.scitotenv.2024.174348

[22] Page MJ, McKenzie JE, Bossuyt PM, et al. The PRISMA 2020 statement: an updated guideline for reporting systematic reviews. BMJ. 2021;372:n71. 33782057 10.1136/bmj.n71 PMC8005924

[23] Lo CK, Mertz DLM. Newcastle-Ottawa scale: comparing reviewers’ to authors’ assessments. BMC Med Res Methodol. 2014;14:45. 24690082 10.1186/1471-2288-14-45 PMC4021422

[24] Gu W, Xie D, Li Q, et al. Association of humidity and precipitation with asthma: a systematic review and meta-analysis. Front Allergy. 2024;5:1483430. 39713043 10.3389/falgy.2024.1483430 PMC11659254

[25] Mustafic H, Jabre P, Caussin C, et al. Main air pollutants and myocardial infarction: a systematic review and meta-analysis. JAMA. 2012;307(7):713–21. 22337682 10.1001/jama.2012.126

[26] Higgins JPT, Morgan RL, Rooney AA, et al. A tool to assess risk of bias in non-randomized follow-up studies of exposure effects (ROBINS-E). Environ Int. 2024;186:108602. 38555664 10.1016/j.envint.2024.108602 PMC11098530

[27] Khreis H, Kelly C, Tate J, et al. Exposure to traffic-related air pollution and risk of development of childhood asthma: a systematic review and meta-analysis. Environ Int. 2017;100:1–31. 27881237 10.1016/j.envint.2016.11.012

[28] Davies HT, Crombie IK, Tavakoli M. When can odds ratios mislead? BMJ. 1998;316(7136):989–91. 9550961 10.1136/bmj.316.7136.989 PMC1112884

[29] Jia P, Luo M, Li Y, et al. Fast-food restaurant, unhealthy eating, and childhood obesity: a systematic review and meta-analysis. Obes Rev. 2021;22(Suppl 1):e12944. 31507064 10.1111/obr.12944 PMC7988557

[30] Liu J, Varghese BM, Hansen A, et al. Is there an association between hot weather and poor mental health outcomes? A systematic review and meta-analysis. Environ Int. 2021;153:106533. 33799230 10.1016/j.envint.2021.106533

[31] Peel MC, McMahon BLFTA. Updated world map of the Köppen-Geiger climate classification. Hydrol Earth Syst Sci. 2007;11(5):1633–44.

[32] Phua J, Joynt GM, Nishimura M, et al. Withholding and withdrawal of life-sustaining treatments in low-middle-income versus high-income Asian countries and regions. Intensive Care Med. 2016;42(7):1118–27. 27071388 10.1007/s00134-016-4347-y

[33] Faridi S, Allen RW, Brook RD, et al. An updated systematic review and meta-analysis on portable air cleaners and blood pressure: recommendations for users and manufacturers. Ecotoxicol Environ Saf. 2023;263:115227. 37421892 10.1016/j.ecoenv.2023.115227

[34] Faridi S, Brook RD, Yousefian F, et al. Effects of respirators to reduce fine particulate matter exposures on blood pressure and heart rate variability: a systematic review and meta-analysis. Environ Pollut. 2022;303:119109. 35271952 10.1016/j.envpol.2022.119109 PMC10411486

[35] Liang W, Zhu H, Xu J, et al. Ambient air pollution and gestational diabetes mellitus: an updated systematic review and meta-analysis. Ecotoxicol Environ Saf. 2023;255:114802. 36934545 10.1016/j.ecoenv.2023.114802

[36] Ai S, Liu L, Xue Y, et al. Prenatal exposure to air pollutants associated with allergic diseases in children: which pollutant, when exposure, and what disease? A systematic review and meta-analysis. Clin Rev Allergy Immunol. 2024;66(2):149–63. 38639856 10.1007/s12016-024-08987-3

[37] Guyatt G, Oxman AD, Akl EA, et al. GRADE guidelines: 1. Introduction-GRADE evidence profiles and summary of findings tables. J Clin Epidemiol. 2011;64(4):383–94. 21195583 10.1016/j.jclinepi.2010.04.026

[38] Hughes AM, Lucas RM, Ponsonby AL, et al. The role of latitude, ultraviolet radiation exposure and vitamin D in childhood asthma and hayfever: an Australian multicenter study. Pediatr Allergy Immunol. 2011;22(3):327–33. 20880353 10.1111/j.1399-3038.2010.01099.x

[39] Hsieh SP, Hsieh CJ, Tseng CC, et al. Allergic rhinitis: association with air pollution and weather changes, and comparison with that of allergic conjunctivitis in Taiwan. Atmosphere. 2020;11(11):11.

[40] Duan J, Wang X, Zhao D, et al. Risk effects of high and low relative humidity on allergic rhinitis: time series study. Environ Res. 2019;173:373–8. 30954910 10.1016/j.envres.2019.03.040

[41] Weiland SK, Hüsing A, Strachan DP, et al. Climate and the prevalence of symptoms of asthma, allergic rhinitis, and atopic eczema in children. Occup Environ Med. 2004;61(7):609–15. 15208377 10.1136/oem.2002.006809 PMC1740799

[42] Zanolin ME, Pattaro C, Corsico A, et al. The role of climate on the geographic variability of asthma, allergic rhinitis and respiratory symptoms: results from the Italian study of asthma in young adults. Allergy. 2004;59(3):306–14. 14982513 10.1046/j.1398-9995.2003.00391.x

[43] Qiu C, Feng W, An X, et al. The effect of fine particulate matter exposure on allergic rhinitis of adolescents aged 10–13 years: a cross-sectional study from Chongqing, China. Front Public Health. 2022;10:921089. 36388289 10.3389/fpubh.2022.921089 PMC9642846

[44] Kurt E, Metintas S, Basyigit I, et al. Prevalence and risk factors of allergies in Turkey: results of a multicentric cross-sectional study in children. Pediatr Allergy Immunol. 2007;18(7):566–74. 18001428 10.1111/j.1399-3038.2007.00551.x

[45] Gao J, Lu M, Sun Y, et al. Changes in ambient temperature increase hospital outpatient visits for allergic rhinitis in Xinxiang, China. BMC Public Health. 2021;21(1):600. 33771145 10.1186/s12889-021-10671-6 PMC8004401

[46] Breton MC, Garneau M, Fortier I, et al. Relationship between climate, pollen concentrations of *Ambrosia* and medical consultations for allergic rhinitis in Montreal, 1994–2002. Sci Total Environ. 2006;370(1):39–50. 16899280 10.1016/j.scitotenv.2006.05.022

[47] Todkill D, de Jesus Colon Gonzalez F, Morbey R, et al. Environmental factors associated with general practitioner consultations for allergic rhinitis in London, England: a retrospective time series analysis. BMJ Open. 2020;10(12):e036724. 33277274 10.1136/bmjopen-2019-036724 PMC7722376

[48] Bhattacharyya N. Does annual temperature influence the prevalence of otolaryngologic respiratory diseases? Laryngoscope. 2009;119(10):1882–6. 19598211 10.1002/lary.20613

[49] Kim SH, Park HS, Jang JY. Impact of meteorological variation on hospital visits of patients with tree pollen allergy. BMC Public Health. 2011;11:890. 22115497 10.1186/1471-2458-11-890 PMC3315442

[50] Upperman CR, Parker JD, Akinbami LJ, et al. Exposure to extreme heat events is associated with increased hay fever prevalence among nationally representative sample of US adults: 1997–2013. J Allergy Clin Immunol Pract. 2017;5(2):435–e441432. 27840238 10.1016/j.jaip.2016.09.016 PMC5346329

[51] He S, Mou Z, Peng L, et al. Impacts of meteorological and environmental factors on allergic rhinitis in children. Int J Biometeorol. 2017;61(5):797–806. 27778095 10.1007/s00484-016-1257-1

[52] Niu Z, Zhang L, Zhang X, et al. Association between air temperature exposure and childhood rhinitis risk, and the mediating role of ambient O3: a multi-city study of 40,103 Chinese preschool children. Sustain Cities Soc. 2025;119:106122.

[53] Wang Y, Zhong W, Wang Z, et al. Effects of meteorological factors and air pollution on the number of allergic rhinitis outpatient visits in Changchun city, China. Sci Rep. 2025;15(1):32406. 41087446 10.1038/s41598-025-16265-1 PMC12521511

[54] Choi YJ, Lee KS, Oh JW. The impact of climate change on pollen season and allergic sensitization to pollens. Immunol Allergy Clin North Am. 2021;41(1):97–109. 33228876 10.1016/j.iac.2020.09.004

[55] Freye HB, King J, Litwin CM. Variations of pollen and mold concentrations in 1998 during the strong El Niño event of 1997–1998 and their impact on clinical exacerbations of allergic rhinitis, asthma, and sinusitis. Allergy Asthma Proc. 2001;22(4):239–247. 11552675

[56] Naeem M, Manzoor S, Abid MU, et al. Fungal proteases as emerging biocatalysts to meet the current challenges and recent developments in biomedical therapies: an updated review. J Fungi. 2022;8(2):109. 10.3390/jof8020109 PMC8875690 35205863

[57] Anderegg WRL, Abatzoglou JT, Anderegg LDL, et al. Anthropogenic climate change is worsening North American pollen seasons. Proc Natl Acad Sci U S A. 2021;118(7):e2013284118. 33558232 10.1073/pnas.2013284118 PMC7896283

[58] Lee YZ, Kow ASF, Jacquet A, et al. House dust mite allergy in Malaysia: review of research gaps in the current scenario and the way forward. Exp Appl Acarol. 2023;91(4):509–39. 37995026 10.1007/s10493-023-00857-5

[59] Gan H, Luo W, Huang Z, et al. House dust mite components sensitization profile in China, a multi-centre study. Clin Exp Allergy. 2023;53(2):226–9. 36519672 10.1111/cea.14255

[60] Çelebi Sözener Z, Treffeisen ER, Özdel Öztürk B, et al. Global warming and implications for epithelial barrier disruption and respiratory and dermatologic allergic diseases. J Allergy Clin Immunol. 2023;152(5):1033–46. 37689250 10.1016/j.jaci.2023.09.001 PMC10864040

[61] Anderson HM, Wood RA, Busse WW. Dust mite-induced perennial allergic rhinitis in pediatric patients and sublingual immunotherapy. J Allergy Clin Immunol Pract. 2017;5(1):46–51. 27665384 10.1016/j.jaip.2016.07.013

[62] Sun Y, Cui L, Hou J, et al. Role of ventilation and cleaning for controlling house dust mite allergen infestation: a study on associations of house dust mite allergen concentrations with home environment and life styles in Tianjin area, China. Indoor Air. 2022;32(8):e13084. 36040279 10.1111/ina.13084

[63] Han A, Deng S, Yu J, et al. Asthma triggered by extreme temperatures: from epidemiological evidence to biological plausibility. Environ Res. 2023;216(Pt 2):114489. 36208788 10.1016/j.envres.2022.114489

[64] Bouchama A, Aziz MA, Mahri SA, et al. A model of exposure to extreme environmental heat uncovers the human transcriptome to heat stress. Sci Rep. 2017;7(1):9429. 28842615 10.1038/s41598-017-09819-5 PMC5573409

[65] Pacheco SE, Guidos-Fogelbach G, Annesi-Maesano I, et al. Climate change and global issues in allergy and immunology. J Allergy Clin Immunol. 2021;148(6):1366–77. 34688774 10.1016/j.jaci.2021.10.011

[66] Deng L, Ma P, Wu Y, et al. High and low temperatures aggravate airway inflammation of asthma: evidence in a mouse model. Environ Pollut. 2020;256:113433. 31761597 10.1016/j.envpol.2019.113433

[67] Schreurs W, Schermer TRJ, Akkermans RP, et al. 25-year retrospective longitudinal study on seasonal allergic rhinitis associations with air temperature in general practice. NPJ Prim Care Respir Med. 2022;32(1):54. 36473873 10.1038/s41533-022-00319-2 PMC9723707

[68] Domingo KN, Gabaldon KL, Hussari MN, et al. Impact of climate change on paediatric respiratory health: pollutants and aeroallergens. Eur Respir Rev. 2024;33(172):230249. 39009406 10.1183/16000617.0249-2023 PMC11262702

[69] Basu R, Pearson D, Malig B, et al. The effect of high ambient temperature on emergency room visits. Epidemiology. 2012;23(6):813–20. 23007039 10.1097/EDE.0b013e31826b7f97

[70] Austin CR, Wanzek J, Scammacca NK, et al. The relationship between study quality and the effects of supplemental reading interventions: a meta-analysis. Except Child. 2019;85(3):347–66. 31588147 10.1177/0014402918796164 PMC6777867

[71] Wong E, Backholer K, Gearon E, et al. Diabetes and risk of physical disability in adults: a systematic review and meta-analysis. Lancet Diabetes Endocrinol. 2013;1(2):106–14. 24622316 10.1016/S2213-8587(13)70046-9

[72] Huang H, Ye R, Qi M, et al. Wind-mediated horseweed (*Conyza canadensis*) gene flow: pollen emission, dispersion, and deposition. Ecol Evol. 2015;5(13):2646–58. 26257877 10.1002/ece3.1540 PMC4523360

[73] Sofiev M, Siljamo P, Ranta H, et al. A numerical model of Birch pollen emission and dispersion in the atmosphere. Description of the emission module. Int J Biometeorol. 2013;57(1):45–58. 22410824 10.1007/s00484-012-0532-z PMC3527742

[74] Jones AM, Harrison RM. The effects of meteorological factors on atmospheric bioaerosol concentrations-a review. Sci Total Environ. 2004;326(1–3):151–80. 15142773 10.1016/j.scitotenv.2003.11.021

[75] D’Amato GCL. Effects of climate change on environmental factors in respiratory allergic diseases. Clin Exp Allergy. 2008;38(8):1264–74. 18537982 10.1111/j.1365-2222.2008.03033.x

[76] Guarnieri G, Olivieri B, Senna G, et al. Relative humidity and its impact on the immune system and infections. Int J Mol Sci. 2023;24(11):9456. 10.3390/ijms24119456 PMC10253274 37298409

[77] Desrosiers M, Baroody FM, Proud D, et al. Treatment with hot, humid air reduces the nasal response to allergen challenge. J Allergy Clin Immunol. 1997;99(1 Pt 1):77–86. 9003214 10.1016/s0091-6749(97)70303-8

[78] Assanasen P, Baroody FM, Naureckas E, et al. Hot, humid air increases cellular influx during the late-phase response to nasal challenge with antigen. Clin Exp Allergy. 2001;31(12):1913–22. 11737044 10.1046/j.1365-2222.2001.01271.x

[79] Farbotko C, Waitt G. Residential air-conditioning and climate change: voices of the vulnerable. Health Promot J Austr. 2011;22(4):13–5. 22518913

[80] Soneja S, Jiang C, Fisher J, et al. Exposure to extreme heat and precipitation events associated with increased risk of hospitalization for asthma in Maryland, U.S.A. Environ Health. 2016;15:57. 27117324 10.1186/s12940-016-0142-z PMC4847234

[81] D’Amato G, Liccardi G, Frenguelli G. Thunderstorm-asthma and pollen allergy. Allergy. 2007;62(1):11–6. 17156336 10.1111/j.1398-9995.2006.01271.x

