# Supplementary material for: Association of meteorological factors with allergic rhinitis: a systematic review and meta-analysis
Source: BMC Public Health. 2025 Dec 30;26:409. doi: 10.1186/s12889-025-26078-6 (PMC12860023; doi:10.1186/s12889-025-26078-6)
Supplement: Supplementary file 2 — Supplementary Material 2: Table S1 Search Strategies for Each Database. Table S2 Quality assessment of time-series analysis and case-crossover studies. Table S3 Quality assessment of ecological and cross-sectional studies using the Newcastle-Ottawa-Scale. Table S4 Quality assessment of case-control studies using the Newcastle-Ottawa-Scale. Table S5 GRADE certainty of evidence rating and rationale. [file 12889_2025_26078_MOESM2_ESM.docx]

**Table S1** Search Strategies for Each Database

| **Database** | **Search string** | **Results** |
| --- | --- | --- |
| PubMed | (("allergic rhinitis"[Title/Abstract] OR "hay fever"[Title/Abstract] OR "nasal allergy"[Title/Abstract] OR "pollen allergy"[Title/Abstract] OR "perennial allergic rhinitis"[Title/Abstract] OR "seasonal allergic rhinitis"[Title/Abstract] OR "Pollinosis"[Title/Abstract] OR "rhinitis"[Title/Abstract]) AND ("climate"[Title/Abstract] OR "weather"[Title/Abstract] OR "meteorolog*"[Title/Abstract] OR "climate change"[Title/Abstract] OR "temperature"[Title/Abstract] OR "humidity"[Title/Abstract] OR "rain*"[Title/Abstract] OR "precipitation"[Title/Abstract] OR "atmospheric pressure"[Title/Abstract] OR "wind speed"[Title/Abstract] OR "sunshine duration"[Title/Abstract])) | 1169 |
| Embase | ('allergic rhinitis':ab,ti OR 'hay fever':ab,ti OR 'nasal allergy':ab,ti OR 'pollen allergy':ab,ti OR 'perennial allergic rhinitis':ab,ti OR 'seasonal allergic rhinitis':ab,ti OR pollinosis:ab,ti OR rhinitis:ab,ti) AND (climate:ab,ti OR weather:ab,ti OR meteorolog*:ab,ti OR 'climate change':ab,ti OR temperature:ab,ti OR humidity:ab,ti OR rain*:ab,ti OR precipitation:ab,ti OR 'atmospheric pressure':ab,ti OR 'wind speed':ab,ti OR 'sunshine duration':ab,ti) | 1836 |
| Scopus | ( TITLE-ABS-KEY ( "allergic rhinitis" ) OR TITLE-ABS-KEY ( "hay fever" ) OR TITLE-ABS-KEY ( "nasal allergy" ) OR TITLE-ABS-KEY ( "pollen allergy" ) OR TITLE-ABS-KEY ( "perennial allergic rhinitis" ) OR TITLE-ABS-KEY ( "seasonal allergic rhinitis" ) OR TITLE-ABS-KEY ( pollinosis ) OR TITLE-ABS-KEY ( rhinitis) ) AND ( TITLE-ABS-KEY ( climate ) OR TITLE-ABS-KEY ( weather ) OR TITLE-ABS-KEY ( meteorolog* ) OR TITLE-ABS-KEY ( "climate change" ) OR TITLE-ABS-KEY ( temperature ) OR TITLE-ABS-KEY ( humidity ) OR TITLE-ABS-KEY ( rain* ) OR TITLE-ABS-KEY ( precipitation ) OR TITLE-ABS-KEY ( "atmospheric pressure" ) OR TITLE-ABS-KEY ( "wind speed" ) OR TITLE-ABS-KEY ( "sunshine duration" ) ) | 3564 |
| Proquest | (subject("Allergic rhinitis ") OR subject("hay fever") OR subject("nasal allergy") OR subject("pollen allergy") OR subject("perennial allergic rhinitis") OR subject("seasonal allergic rhinitis") OR subject(Pollinosis) OR subject(rhinitis)) AND (subject(climate) OR subject(weather) OR subject(meteorolog*) OR subject("climate change") OR subject(temperature) OR subject(humidity) OR subject("rain*") OR subject(precipitation) OR subject("atmospheric pressure") OR subject("wind speed") OR subject "sunshine duration") | 2426 |
| Web of Science | (((((((TS=("allergic rhinitis" )) OR TS=("hay fever")) OR TS=("nasal allergy")) OR TS=("pollen allergy")) OR TS=("perennial allergic rhinitis")) OR TS=("seasonal allergic rhinitis")) OR TS=(Pollinosis)) OR TS=(rhinitis) AND (((((((((TS=(climate)) OR TS=(weather)) OR TS=("climate change")) OR TS=(temperature)) OR TS=(humidity)) OR TS=("rain*")) OR TS=(precipitation)) OR TS=("atmospheric pressure")) OR TS=("wind speed")) OR TS=("sunshine duration") | 1886 |

**Table S2** Quality assessment of time-series analysis and case-crossover studies

| **First Author** | **Year** | **Allergic rhinitis diagnosis (0 to 1 point)** | **Quality of climate exposure level (0 to 1 point)** | **Adjustment for confounders (0 to 3 point)** | **Score** |
| --- | --- | --- | --- | --- | --- |
| Hsieh S | 2020 | 1 | 1 | 2 | 4/5 |
| Hu Y | 2020 | 1 | 1 | 3 | 5/5 |
| Duan J | 2019 | 1 | 1 | 1 | 3/5 |
| Gao J | 2021 | 1 | 1 | 2 | 4/5 |
| Breton MC | 2006 | 1 | 1 | 1 | 3/5 |
| Todkill D | 2020 | 1 | 1 | 3 | 5/5 |
| Kim SH | 2011 | 1 | 1 | 1 | 3/5 |
| Wang Y | 2025 | 1 | 1 | 2 | 4/5 |

Since no validated scales of time-series and case-crossover studies were recommended by New Castle Ottawa and Cochrane risk of bias tool, we evaluated the validity based on Mustafic’s study . Three components were assessed, including allergic rhinitis diagnosis (0 to 1 point),Quality of climate exposure level (0 to 1) and adjustment for confounders (0 to 3). climate exposure: Tmperature/Humidity/Wind speed/precipitation/Atmospheric pressure

**Table S3** Quality assessment of ecological and cross-sectional studies using the Newcastle-Ottawa-Scale

| **First Author** | **Year** | **Representativeness of the sample （Max. 1）** | **Sample Size (Max. 1)** | **Non-Respondents  (Max. 1)** | **Ascertainment of Exposure (Max. 2)** | **Comparability （Max.2）** | **Assessment of Outcome  (Max. 2)** | **Statistical Test  (Max. 1)** | **Score** |
| --- | --- | --- | --- | --- | --- | --- | --- | --- | --- |
| Lee YL | 2003 | 1 | 1 | 0 | 2 | 2 | 2 | 1 | 9/10 |
| Wang J | 2021 | 1 | 1 | 0 | 1 | 1 | 1 | 1 | 6/10 |
| Wang XY | 2018 | 1 | 1 | 1 | 2 | 1 | 1 | 1 | 8/10 |
| Qiu C | 2022 | 1 | 1 | 0 | 1 | 1 | 1 | 1 | 6/10 |
| Kurt E | 2007 | 1 | 1 | 0 | 1 | 1 | 1 | 1 | 6/10 |
| Wang J | 2019 | 1 | 1 | 0 | 1 | 1 | 1 | 1 | 6/10 |
| Silverberg JI | 2015 | 1 | 1 | 0 | 1 | 1 | 2 | 1 | 7/10 |
| Bhattacharyya N | 2009 | 1 | 1 | 0 | 2 | 1 | 2 | 1 | 8/10 |
| Weiland SK | 2004 | 1 | 1 | 0 | 1 | 1 | 2 | 1 | 7/10 |
| Zanolin ME | 2004 | 1 | 1 | 0 | 2 | 1 | 1 | 1 | 7/10 |
| Upperman CR | 2017 | 1 | 1 | 0 | 2 | 1 | 2 | 1 | 8/10 |
| He S | 2017 | 1 | 1 | 0 | 1 | 1 | 2 | 1 | 7/10 |
| Niu Z | 2025 | 1 | 1 | 0 | 2 | 2 | 2 | 1 | 9/10 |

|  | | | | | | | | | | |
| --- | --- | --- | --- | --- | --- | --- | --- | --- | --- | --- |
| **Table S4** Quality assessment of case-control studies using the Newcastle-Ottawa-Scale | | | | | | | | | | |
| **First Author** | **Year** | **Is the case definition adequate （Max. 1）** | **Representativeness of the cases （Max. 1）** | **Selection of Controls （Max. 1）** | **Definition of Controls （Max. 1）** | **Comparability of cases and controls on the basis of the design or analysis （Max. 2）** | **Ascertainment of exposure （Max. 1）** | **Same method of ascertainment for cases and controls （Max. 1）** | **Non-Response rate （Max. 1）** | **Score** |
| Hughes AM | 2011 | 1 | 1 | 1 | 1 | 2 | 1 | 1 | 0 | 8/9 |

| **Table S5** GRADE certainty of evidence rating and rationale | | | | | | | | | | |  |  |  |
| --- | --- | --- | --- | --- | --- | --- | --- | --- | --- | --- | --- | --- | --- |
| **Exposure** | **Domains for downgrading** | | | | | |  | **Domains for upgrading** | | |  | **Overall**  **Scores** | **Final certainty assessment** |
|  | **Risk of bias** | **Inconsistency** | **Indirectness** | **Imprecision** | **Publication**  **bias** | |  | **Large effect** | **Does-response** | **Opposing confounding** |  |  |  |
| Temperature | 0 | 0 | 0 | 0 | 0 | |  | 0 | 1 | 0 |  | 1 | High |
|  | Most studies were rated as low/moderate RoB | Substantial heterogeneity existed (I² = 96%), but effect size inconsistencies were primarily from varying study settings. | All studies conducted direct data comparisons for the core research question aligned with PECOS. | Sample sizeswere large  enough. | Funnel plot combined with Egger’s test (P> 0.05) revealed no significant bias | |  | Did not meet the criteria for a large effect | Most studies have observed that the risk of outcomes exhibits an upward trend with increasing temperature. | No clear evidence |  |  |  |
| Humidity | 0 | 0 | 0 | 0 | 0 | |  | 0 | 1 | 0 |  | 1 | High |
|  | Most studies were rated as moderate RoB; | Substantial heterogeneity existed (I² = 98%), but effect size inconsistencies were primarily from varying study settings. | All studies conducted direct data comparisons for the core research question aligned with PECOS. | Sample sizeswere large  enough. | Funnel plot combined with Egger’s test (P> 0.05) revealed no significant bias | |  | Did not meet the criteria for a large effect | Most studies have observed that the risk of outcomes exhibits a downward trend with increasing humidity. | No clear evidence |  |  |  |
| Precipitation | 0 | 0 | 0 | -1 | 0 | |  | 0 | 0 | 0 |  | -1 | Low |
|  | All studieswere ratedas low or moderate RoB. | Substantial heterogeneity existed (I² = 83%), but effect size inconsistencies were primarily from varying study settings. | All studies conducted direct data comparisons for the core research question aligned with PECOS. | The included studies were few, and results were notably affected by a single study | Funnel plot combined with Egger’s test (P> 0.05) revealed no significant bias | |  | Did not meet the criteria for a large effect | Insufficient studies to confirm dose-response | No clear evidence |  |  |  |
| Exposure | Domains for downgrading | | | | | |  | Domains for upgrading | | |  | Overall | Final certainty assessment |
|  | Risk of bias | Inconsistency | Indirectness | Imprecision | Publication  bias | |  | Large effect | Does-response | Opposing confounding |  |  |  |
| Other metrics | 0 | -1 | 0 | -1 | -1 | |  | 0 | 0 | 0 |  | -3 | Very low |
|  | All studieswere ratedas moderate RoB. | A small number of studies renders it unable to adequately assess heterogeneity. | All studies conducted direct data comparisons for the core research question aligned with PECOS | Included studies were few, and 95% CIs encompassed the null effect | | The limited number of included studies may introduce a risk of bias |  | Did not meet the criteria for a large effect | Insufficient studies to confirm dose-response | No clear evidence |  |  |  |
