# Supplementary figures and images for: Association of meteorological factors with allergic rhinitis: a systematic review and meta-analysis
Source: BMC Public Health. 2025 Dec 30;26:409. doi: 10.1186/s12889-025-26078-6 (PMC12860023; doi:10.1186/s12889-025-26078-6)

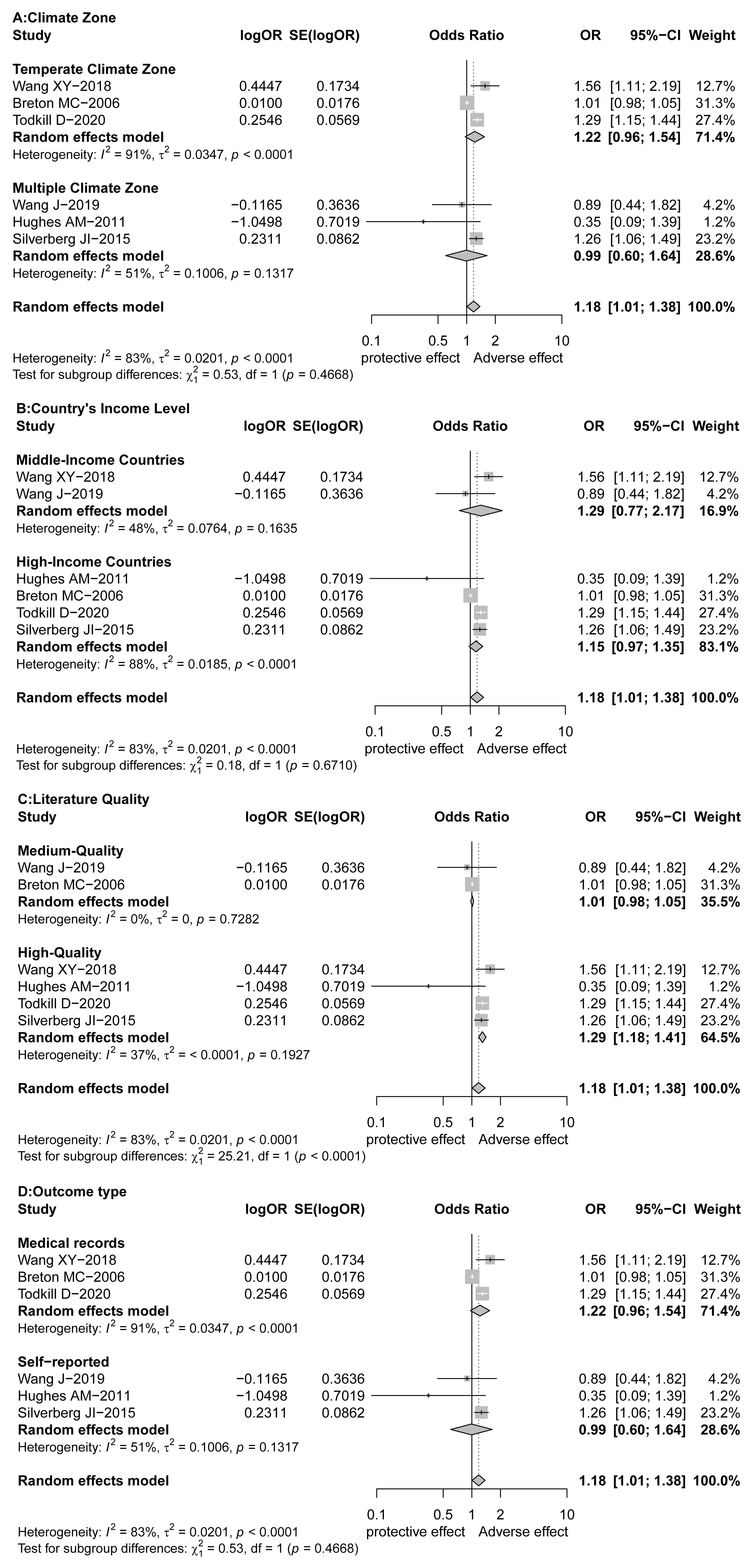

Supplement: Supplementary file 3 — Supplementary Material 3: Fig. S1 Forest plot of the relationship between atmospheric pressure, wind speed and allergic rhinitis. A. Atmospheric pressure; B. Wind speed. Fig. S2 Subgroup analysis of allergic rhinitis and temperature. A. Temperature Measure; B. Climate Zone; C. Country’s Income Level; D. Literature-Quality; E. Outcome type. Fig. S3 Subgroup analysis of between allergic rhinitis and humidity. A. Climate Zone; B. Country’s Income Level; C. Literature-Quality; D. Outcome type. Fig. S4 Subgroup analysis of between allergic rhinitis and Precipitation. A. Climate Zone; B. Country’s Income Level; C. Literature-Quality; D. Outcome type. Fig. S5 Sensitivity analysis of meteorological factors and allergic rhinitis. A. Temperature; B. Humidity; C. Precipitation. Fig. S6 Temperature and allergic rhinitis funnel plot. Fig. S7 Humidity and allergic rhinitis funnel plot. Fig. S8 Precipitation and allergic rhinitis funnel plot. [file 12889_2025_26078_MOESM3_ESM.zip › Fig. S4.tif]

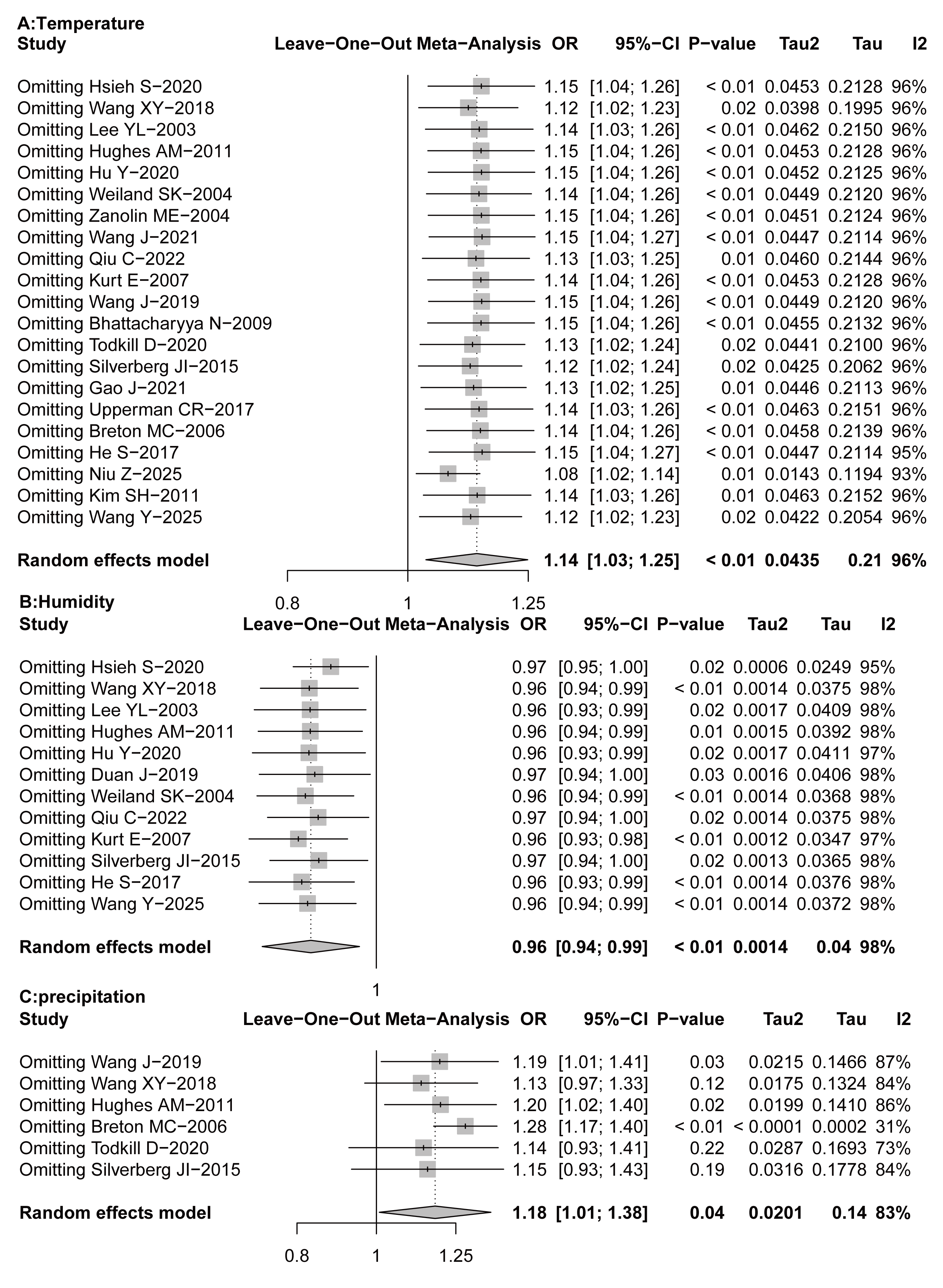

Supplement: Supplementary file 3 — Supplementary Material 3: Fig. S1 Forest plot of the relationship between atmospheric pressure, wind speed and allergic rhinitis. A. Atmospheric pressure; B. Wind speed. Fig. S2 Subgroup analysis of allergic rhinitis and temperature. A. Temperature Measure; B. Climate Zone; C. Country’s Income Level; D. Literature-Quality; E. Outcome type. Fig. S3 Subgroup analysis of between allergic rhinitis and humidity. A. Climate Zone; B. Country’s Income Level; C. Literature-Quality; D. Outcome type. Fig. S4 Subgroup analysis of between allergic rhinitis and Precipitation. A. Climate Zone; B. Country’s Income Level; C. Literature-Quality; D. Outcome type. Fig. S5 Sensitivity analysis of meteorological factors and allergic rhinitis. A. Temperature; B. Humidity; C. Precipitation. Fig. S6 Temperature and allergic rhinitis funnel plot. Fig. S7 Humidity and allergic rhinitis funnel plot. Fig. S8 Precipitation and allergic rhinitis funnel plot. [file 12889_2025_26078_MOESM3_ESM.zip › Fig. S5.tif]

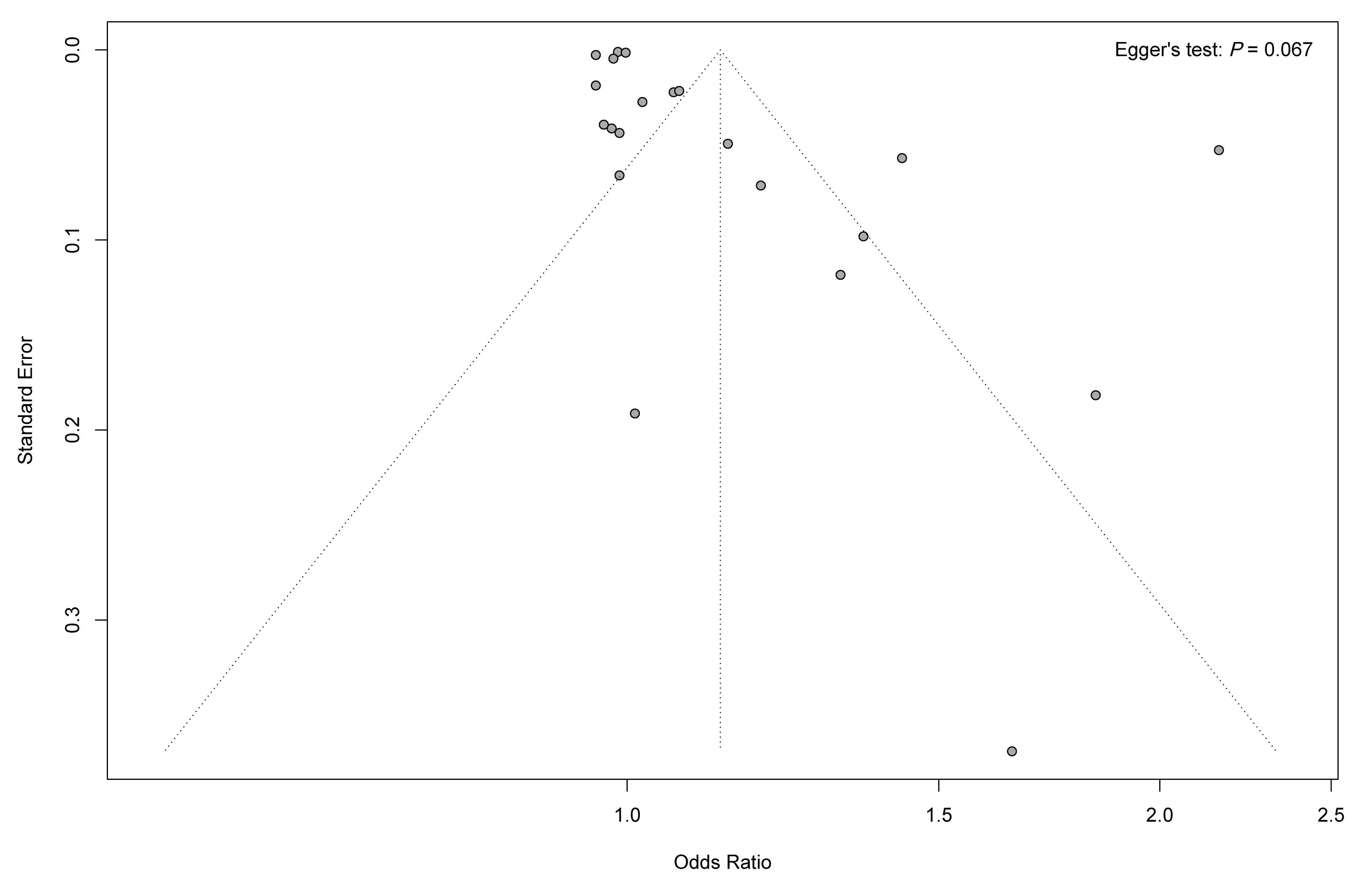

Supplement: Supplementary file 3 — Supplementary Material 3: Fig. S1 Forest plot of the relationship between atmospheric pressure, wind speed and allergic rhinitis. A. Atmospheric pressure; B. Wind speed. Fig. S2 Subgroup analysis of allergic rhinitis and temperature. A. Temperature Measure; B. Climate Zone; C. Country’s Income Level; D. Literature-Quality; E. Outcome type. Fig. S3 Subgroup analysis of between allergic rhinitis and humidity. A. Climate Zone; B. Country’s Income Level; C. Literature-Quality; D. Outcome type. Fig. S4 Subgroup analysis of between allergic rhinitis and Precipitation. A. Climate Zone; B. Country’s Income Level; C. Literature-Quality; D. Outcome type. Fig. S5 Sensitivity analysis of meteorological factors and allergic rhinitis. A. Temperature; B. Humidity; C. Precipitation. Fig. S6 Temperature and allergic rhinitis funnel plot. Fig. S7 Humidity and allergic rhinitis funnel plot. Fig. S8 Precipitation and allergic rhinitis funnel plot. [file 12889_2025_26078_MOESM3_ESM.zip › Fig. S6.tif]

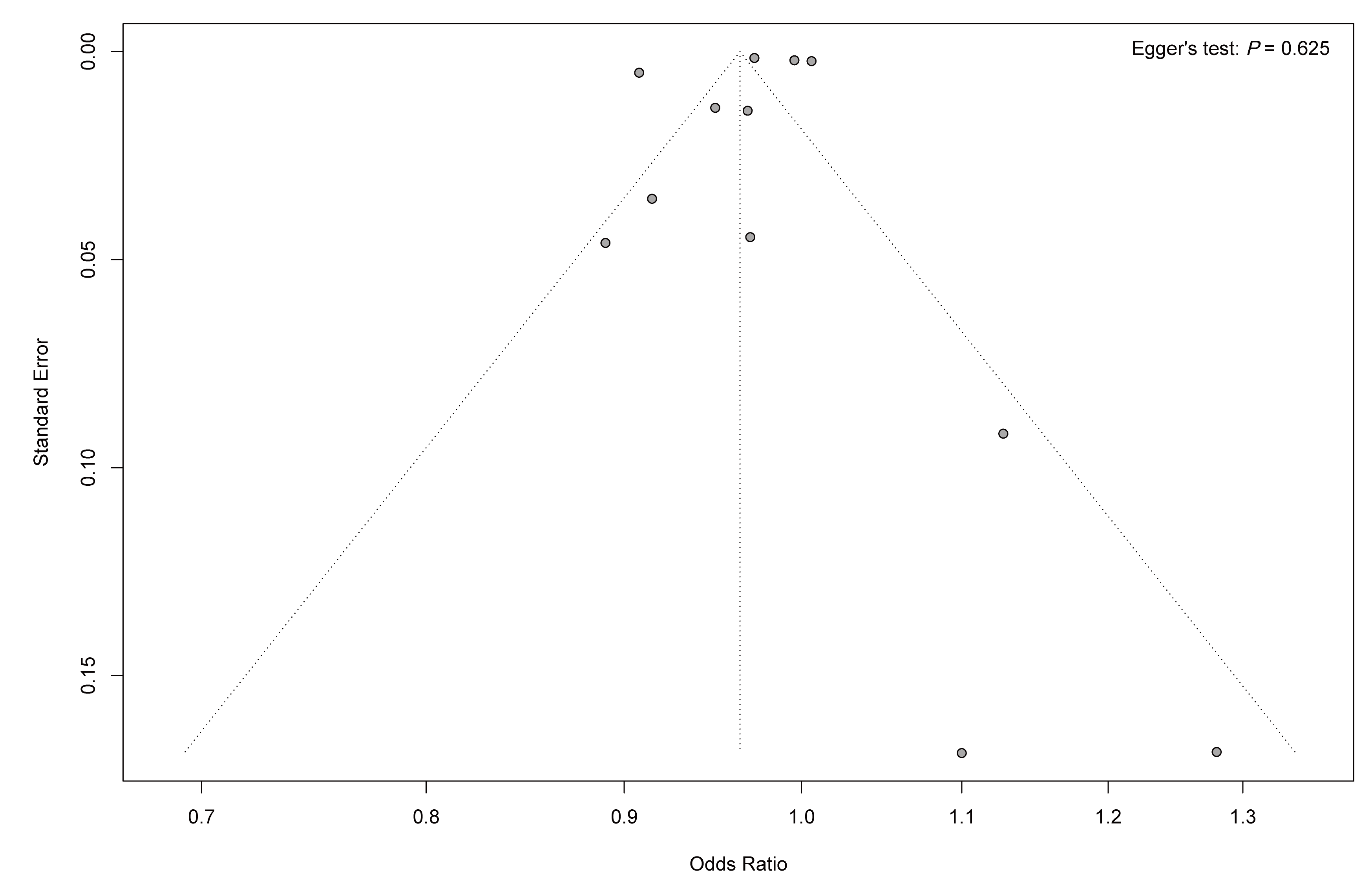

Supplement: Supplementary file 3 — Supplementary Material 3: Fig. S1 Forest plot of the relationship between atmospheric pressure, wind speed and allergic rhinitis. A. Atmospheric pressure; B. Wind speed. Fig. S2 Subgroup analysis of allergic rhinitis and temperature. A. Temperature Measure; B. Climate Zone; C. Country’s Income Level; D. Literature-Quality; E. Outcome type. Fig. S3 Subgroup analysis of between allergic rhinitis and humidity. A. Climate Zone; B. Country’s Income Level; C. Literature-Quality; D. Outcome type. Fig. S4 Subgroup analysis of between allergic rhinitis and Precipitation. A. Climate Zone; B. Country’s Income Level; C. Literature-Quality; D. Outcome type. Fig. S5 Sensitivity analysis of meteorological factors and allergic rhinitis. A. Temperature; B. Humidity; C. Precipitation. Fig. S6 Temperature and allergic rhinitis funnel plot. Fig. S7 Humidity and allergic rhinitis funnel plot. Fig. S8 Precipitation and allergic rhinitis funnel plot. [file 12889_2025_26078_MOESM3_ESM.zip › Fig. S7.tif]

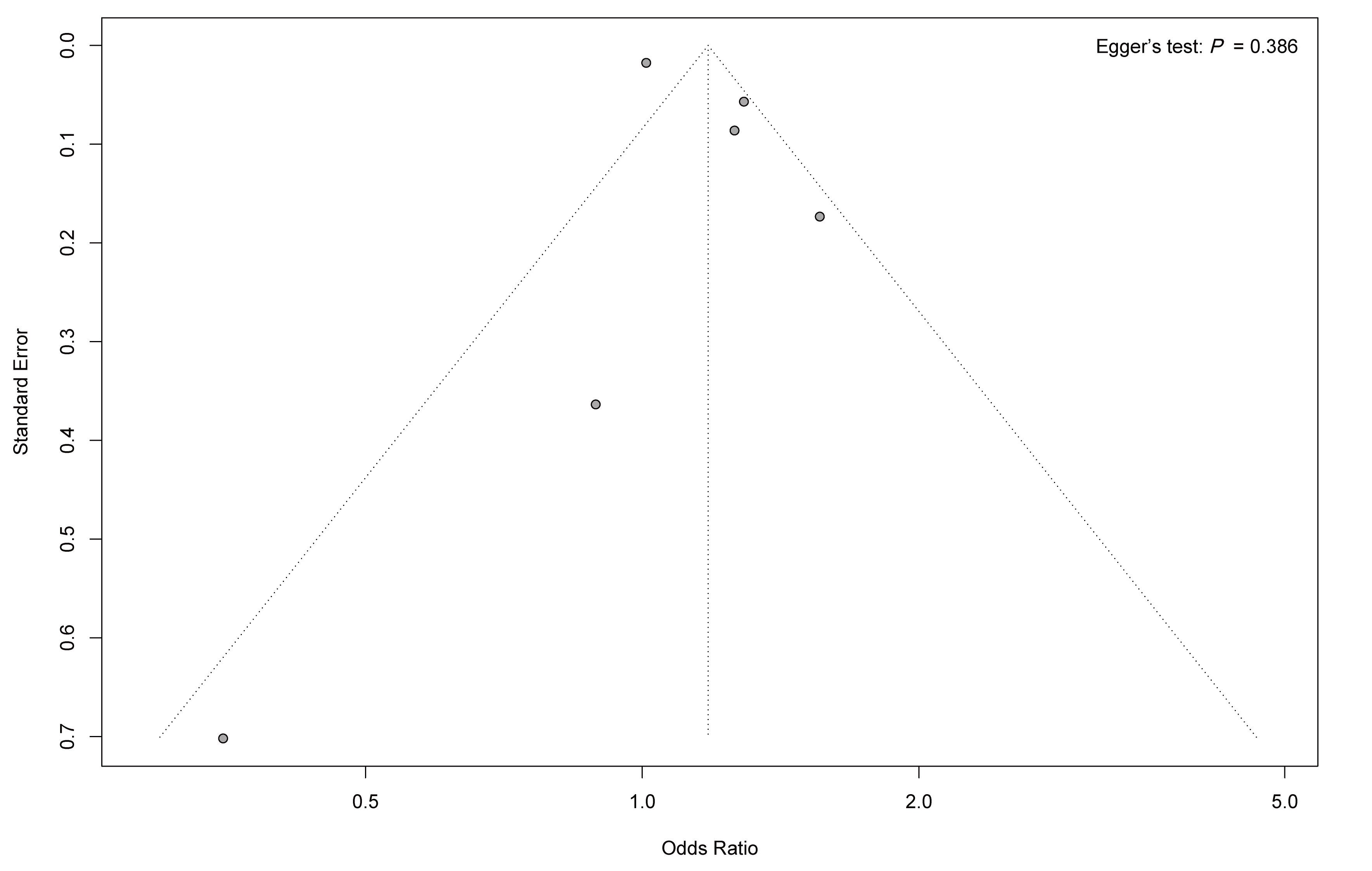

Supplement: Supplementary file 3 — Supplementary Material 3: Fig. S1 Forest plot of the relationship between atmospheric pressure, wind speed and allergic rhinitis. A. Atmospheric pressure; B. Wind speed. Fig. S2 Subgroup analysis of allergic rhinitis and temperature. A. Temperature Measure; B. Climate Zone; C. Country’s Income Level; D. Literature-Quality; E. Outcome type. Fig. S3 Subgroup analysis of between allergic rhinitis and humidity. A. Climate Zone; B. Country’s Income Level; C. Literature-Quality; D. Outcome type. Fig. S4 Subgroup analysis of between allergic rhinitis and Precipitation. A. Climate Zone; B. Country’s Income Level; C. Literature-Quality; D. Outcome type. Fig. S5 Sensitivity analysis of meteorological factors and allergic rhinitis. A. Temperature; B. Humidity; C. Precipitation. Fig. S6 Temperature and allergic rhinitis funnel plot. Fig. S7 Humidity and allergic rhinitis funnel plot. Fig. S8 Precipitation and allergic rhinitis funnel plot. [file 12889_2025_26078_MOESM3_ESM.zip › Fig. S8.tif]

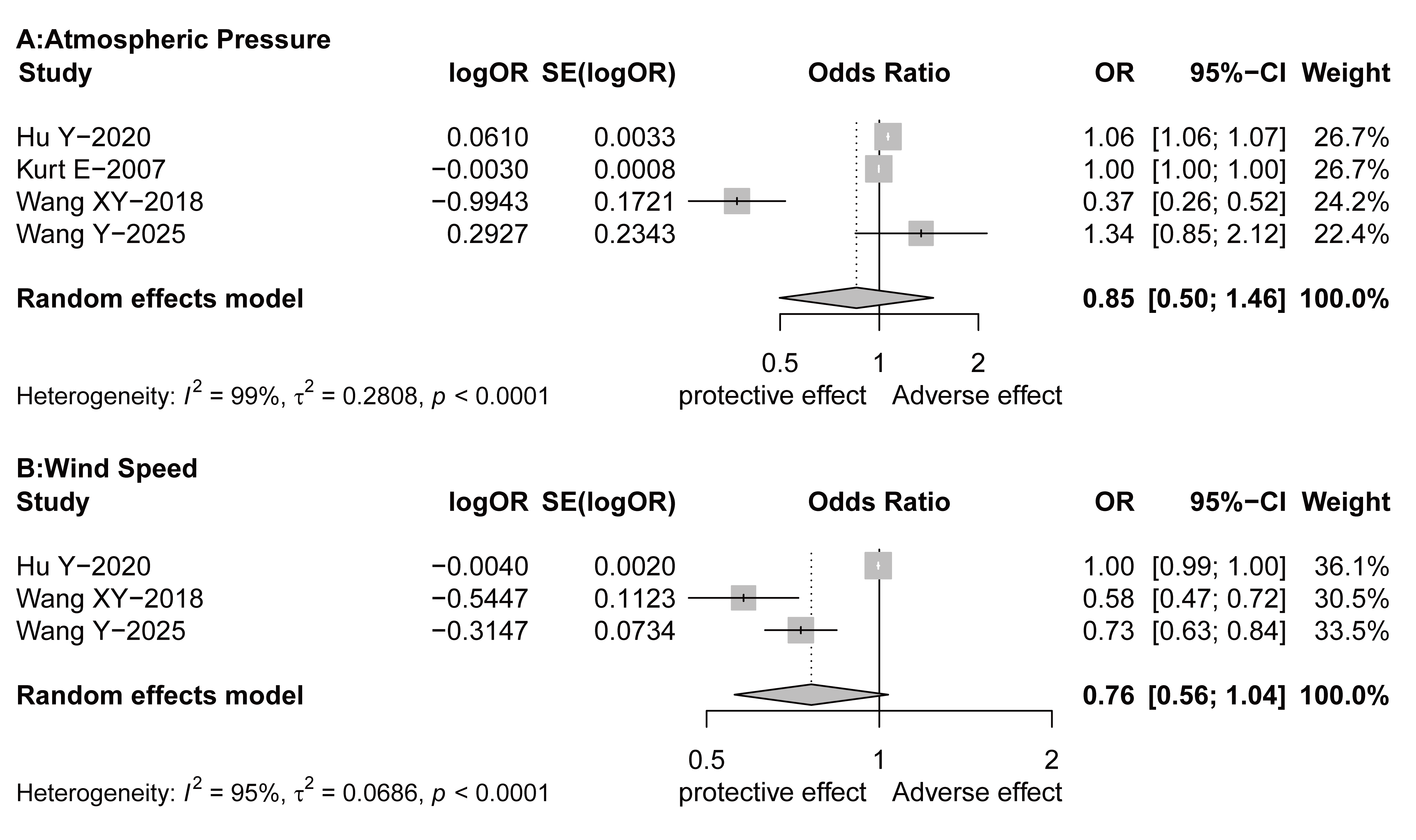

Supplement: Supplementary file 3 — Supplementary Material 3: Fig. S1 Forest plot of the relationship between atmospheric pressure, wind speed and allergic rhinitis. A. Atmospheric pressure; B. Wind speed. Fig. S2 Subgroup analysis of allergic rhinitis and temperature. A. Temperature Measure; B. Climate Zone; C. Country’s Income Level; D. Literature-Quality; E. Outcome type. Fig. S3 Subgroup analysis of between allergic rhinitis and humidity. A. Climate Zone; B. Country’s Income Level; C. Literature-Quality; D. Outcome type. Fig. S4 Subgroup analysis of between allergic rhinitis and Precipitation. A. Climate Zone; B. Country’s Income Level; C. Literature-Quality; D. Outcome type. Fig. S5 Sensitivity analysis of meteorological factors and allergic rhinitis. A. Temperature; B. Humidity; C. Precipitation. Fig. S6 Temperature and allergic rhinitis funnel plot. Fig. S7 Humidity and allergic rhinitis funnel plot. Fig. S8 Precipitation and allergic rhinitis funnel plot. [file 12889_2025_26078_MOESM3_ESM.zip › Fig. S1.tif]

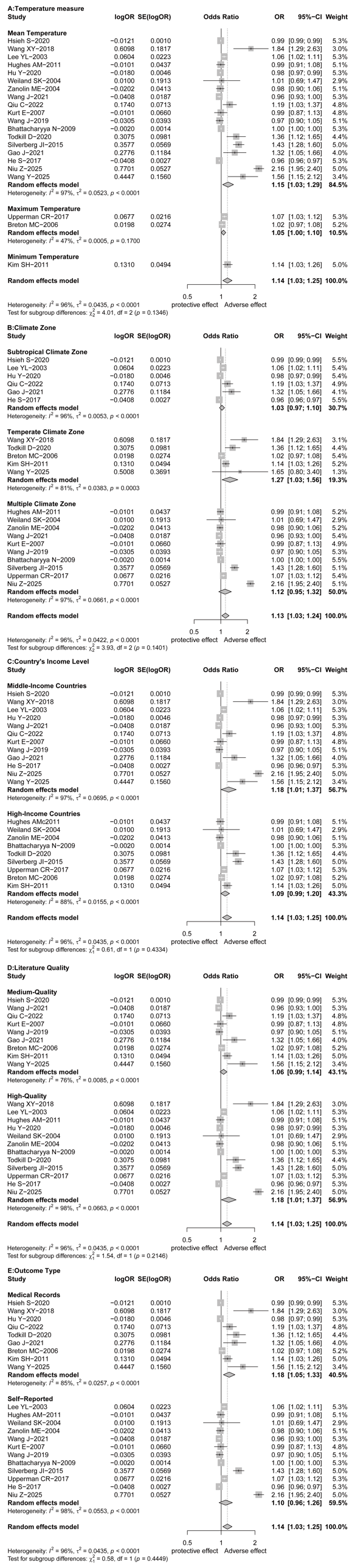

Supplement: Supplementary file 3 — Supplementary Material 3: Fig. S1 Forest plot of the relationship between atmospheric pressure, wind speed and allergic rhinitis. A. Atmospheric pressure; B. Wind speed. Fig. S2 Subgroup analysis of allergic rhinitis and temperature. A. Temperature Measure; B. Climate Zone; C. Country’s Income Level; D. Literature-Quality; E. Outcome type. Fig. S3 Subgroup analysis of between allergic rhinitis and humidity. A. Climate Zone; B. Country’s Income Level; C. Literature-Quality; D. Outcome type. Fig. S4 Subgroup analysis of between allergic rhinitis and Precipitation. A. Climate Zone; B. Country’s Income Level; C. Literature-Quality; D. Outcome type. Fig. S5 Sensitivity analysis of meteorological factors and allergic rhinitis. A. Temperature; B. Humidity; C. Precipitation. Fig. S6 Temperature and allergic rhinitis funnel plot. Fig. S7 Humidity and allergic rhinitis funnel plot. Fig. S8 Precipitation and allergic rhinitis funnel plot. [file 12889_2025_26078_MOESM3_ESM.zip › Fig. S2.tif]

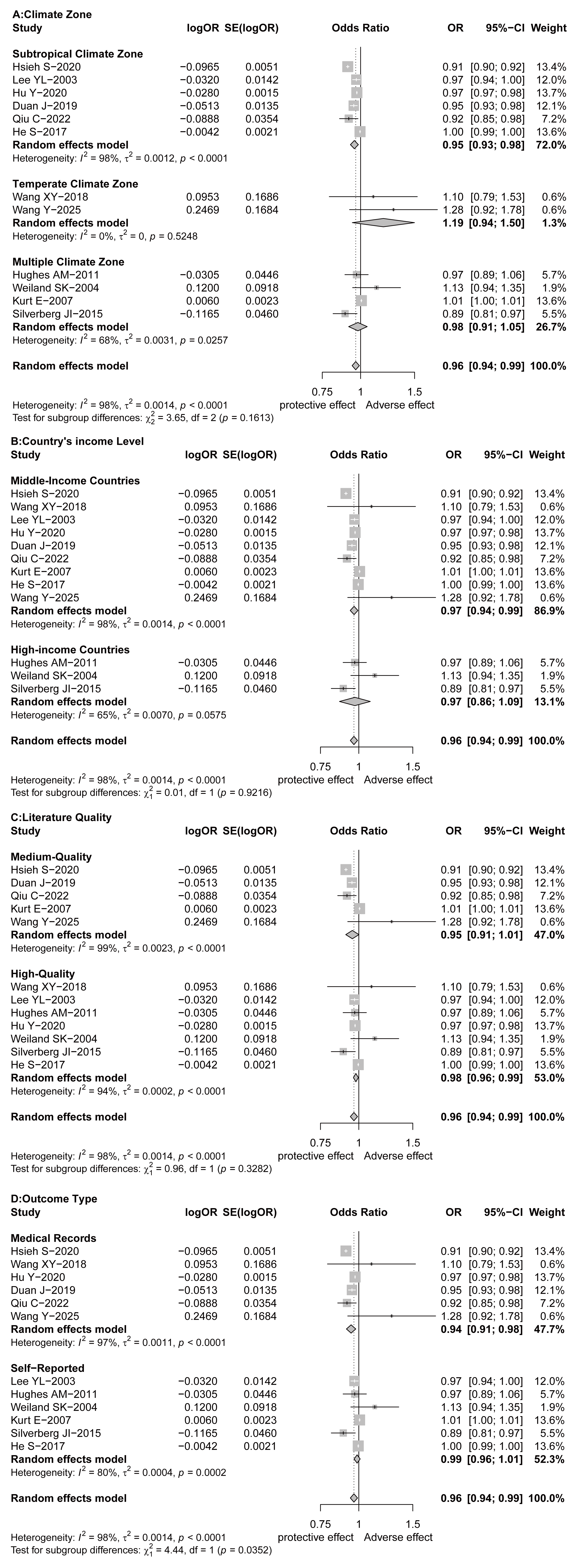

Supplement: Supplementary file 3 — Supplementary Material 3: Fig. S1 Forest plot of the relationship between atmospheric pressure, wind speed and allergic rhinitis. A. Atmospheric pressure; B. Wind speed. Fig. S2 Subgroup analysis of allergic rhinitis and temperature. A. Temperature Measure; B. Climate Zone; C. Country’s Income Level; D. Literature-Quality; E. Outcome type. Fig. S3 Subgroup analysis of between allergic rhinitis and humidity. A. Climate Zone; B. Country’s Income Level; C. Literature-Quality; D. Outcome type. Fig. S4 Subgroup analysis of between allergic rhinitis and Precipitation. A. Climate Zone; B. Country’s Income Level; C. Literature-Quality; D. Outcome type. Fig. S5 Sensitivity analysis of meteorological factors and allergic rhinitis. A. Temperature; B. Humidity; C. Precipitation. Fig. S6 Temperature and allergic rhinitis funnel plot. Fig. S7 Humidity and allergic rhinitis funnel plot. Fig. S8 Precipitation and allergic rhinitis funnel plot. [file 12889_2025_26078_MOESM3_ESM.zip › Fig. S3.tif]
